# Supplementary material for: Poly(fluorenyl aryl piperidinium) membranes and ionomers for anion exchange membrane fuel cells
Source: Nat Commun. 2021 Apr 22;12:2367. doi: 10.1038/s41467-021-22612-3 (PMC8062622; doi:10.1038/s41467-021-22612-3)
Supplement: Supplementary file 1 — Supplementary Information [file 41467_2021_22612_MOESM1_ESM.docx]

Supplementary Information

**Poly(fluorenyl aryl piperidinium) membranes and ionomers for anion exchange membrane fuel cells**

Nanjun Chen^1†^, Ho Hyun Wang^1†^, Sun Pyo Kim^1^, Hae Min Kim^1^, Won Hee Lee^1^, Chuan Hu^1^, Joon Yong Bae^1^, Eun Seob Sim^2^, Yong-Chae Chung^2^, Jue-Hyuk Jang^3^, Sung Jong Yoo^3^, Yongbing Zhuang^4^, Young Moo Lee^1^*

^1^Department of Energy Engineering, College of Engineering, Hanyang University, Seoul 04763, Republic of Korea

^2^Department of Materials Science and Engineering, College of Engineering, Hanyang University, Seoul 04763, Republic of Korea

^3^HydrogenㆍFuel Cell Research Center, Korea Institute of Science and Technology (KIST), Seoul 02792, Republic of Korea

^4^ State Key Laboratory of Biochemical Engineering, Institute of Process Engineering, University of Chinese Academy of Sciences, Chinese Academy of Sciences, Beijing 100190, PR China

Corresponding author: ymlee@hanyang.ac.kr

^†^These authors contributed equally: N. J. Chen and H. H. Wang

**Supplementary Figure 1.** Synthesis routes for PFAP-*x* and PFPN-*x* copolymers.

**
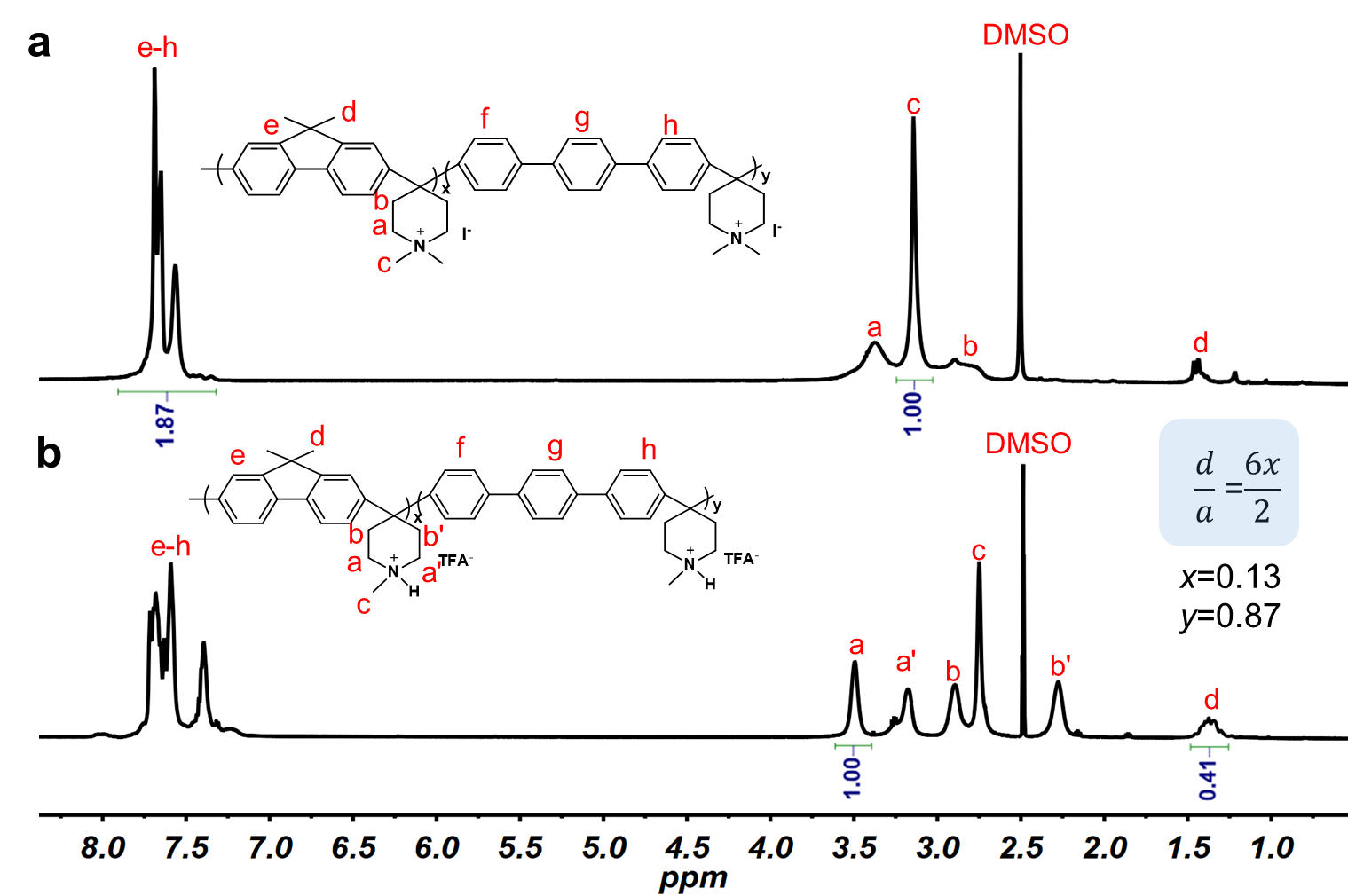
**

**Supplementary Figure 2.** ^1^H NMR spectra of **a**, PFTP-13 and **b**, PFTM-13 with 10% TFA as a co-solvent to eliminate the effect of H_2_O at 3.34 ppm. PFTM-13 is the product before quaternization. TFA was added to DMSO-d_6_ solvent to improve the solubility and eliminate the negative effect of the H_2_O peak (3.34 ppm). The chemical shift of TFA was observed around 13 ppm with a broad peak that was hidden for clarity.


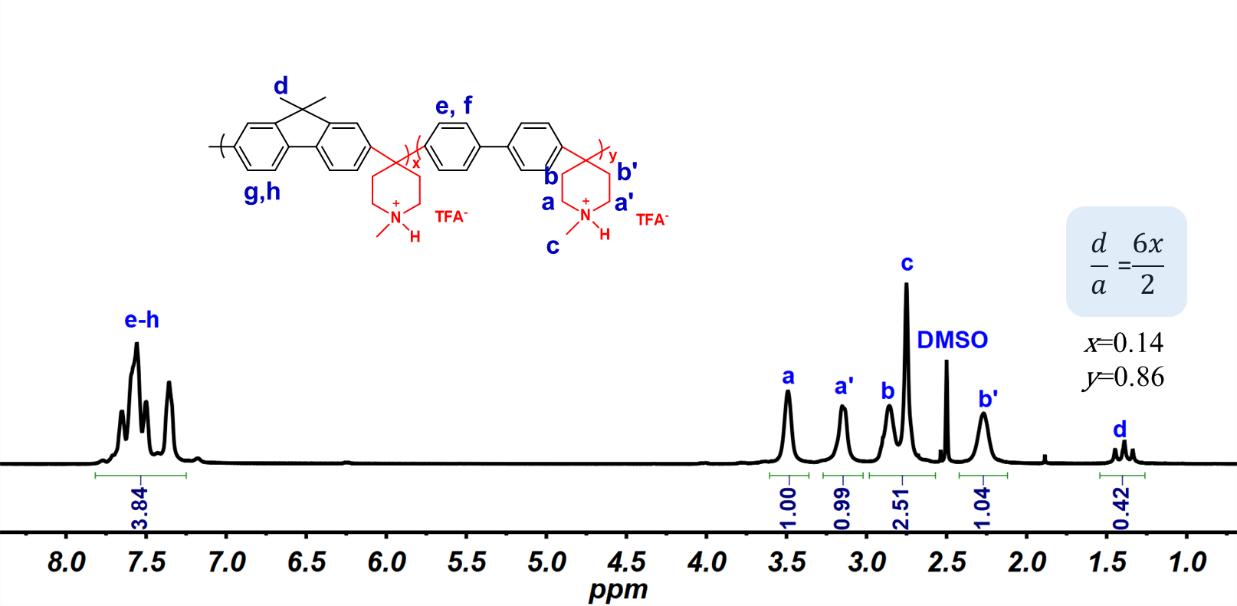


**Supplementary Figure 3.** ^1^H NMR spectrum of PFBM-14 with 10% TFA as a co-solvent in DMSO-d_6_ to eliminate the effect of H_2_O at 3.34 ppm. PFBM-14 is the product before quaternization.


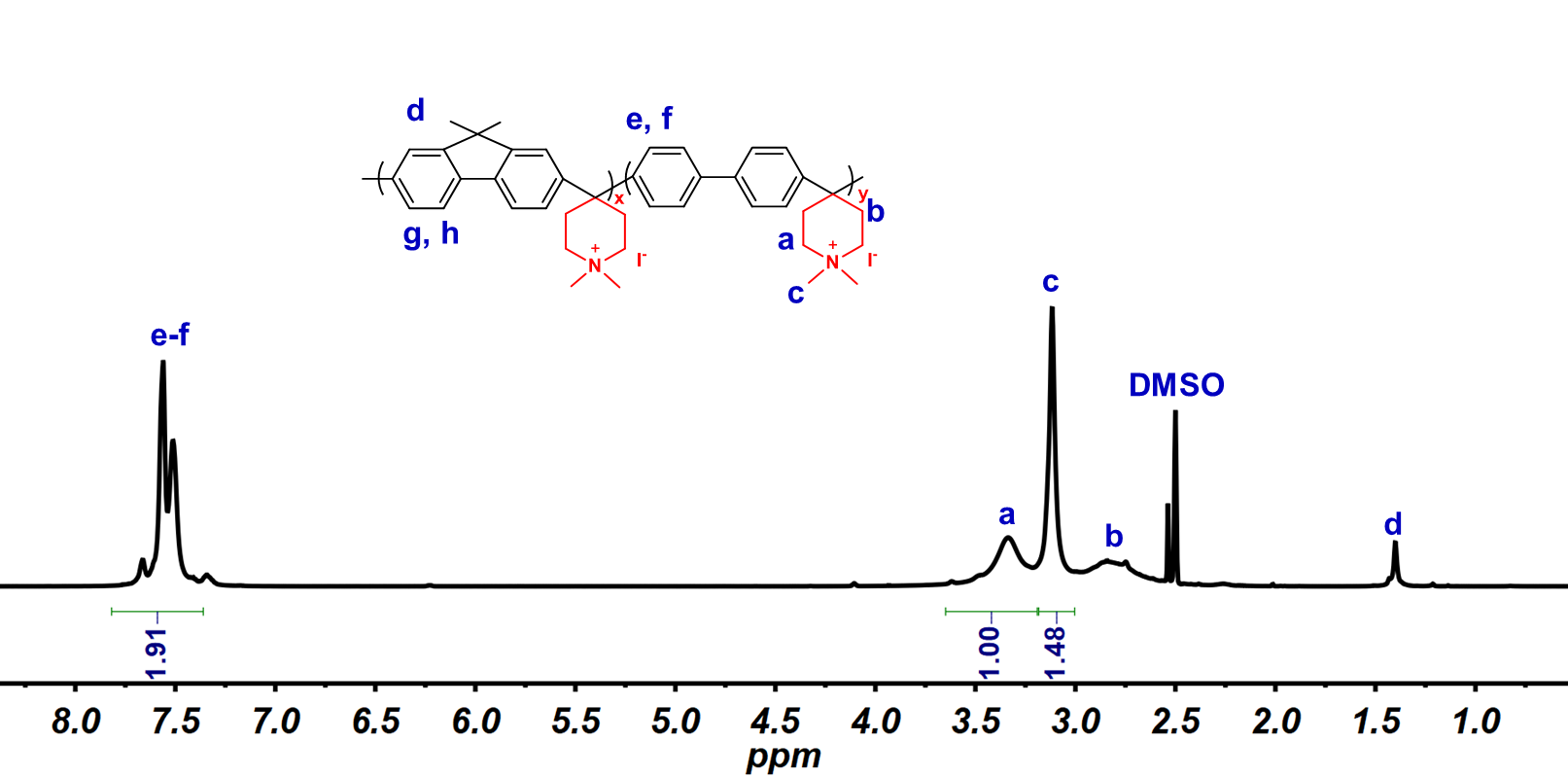


**Supplementary Figure 4.** ^1^H NMR spectrum of PFBP-14 with 10% TFA as a co-solvent in DMSO-d_6_. PFBP-14 is the quaternized product of PFBM-14.


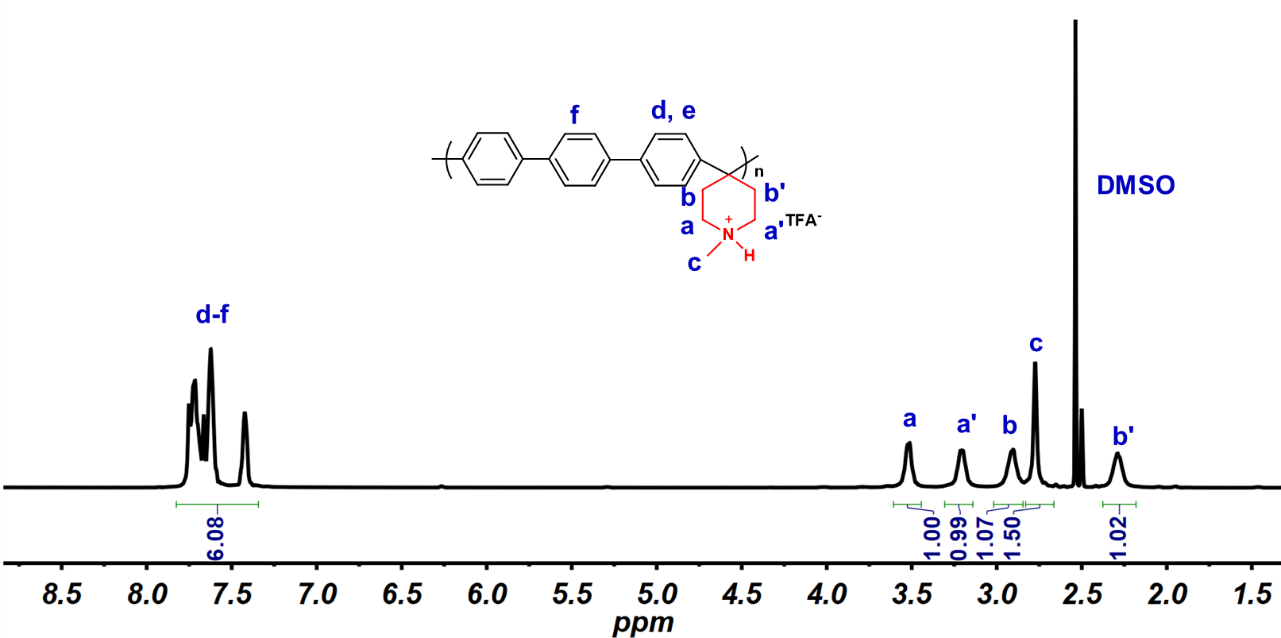


**Supplementary Figure 5.** ^1^H NMR spectrum of PFTM-0 with 10% TFA as a co-solvent in DMSO-d_6_. PFTM-0 is the product before quaternization.


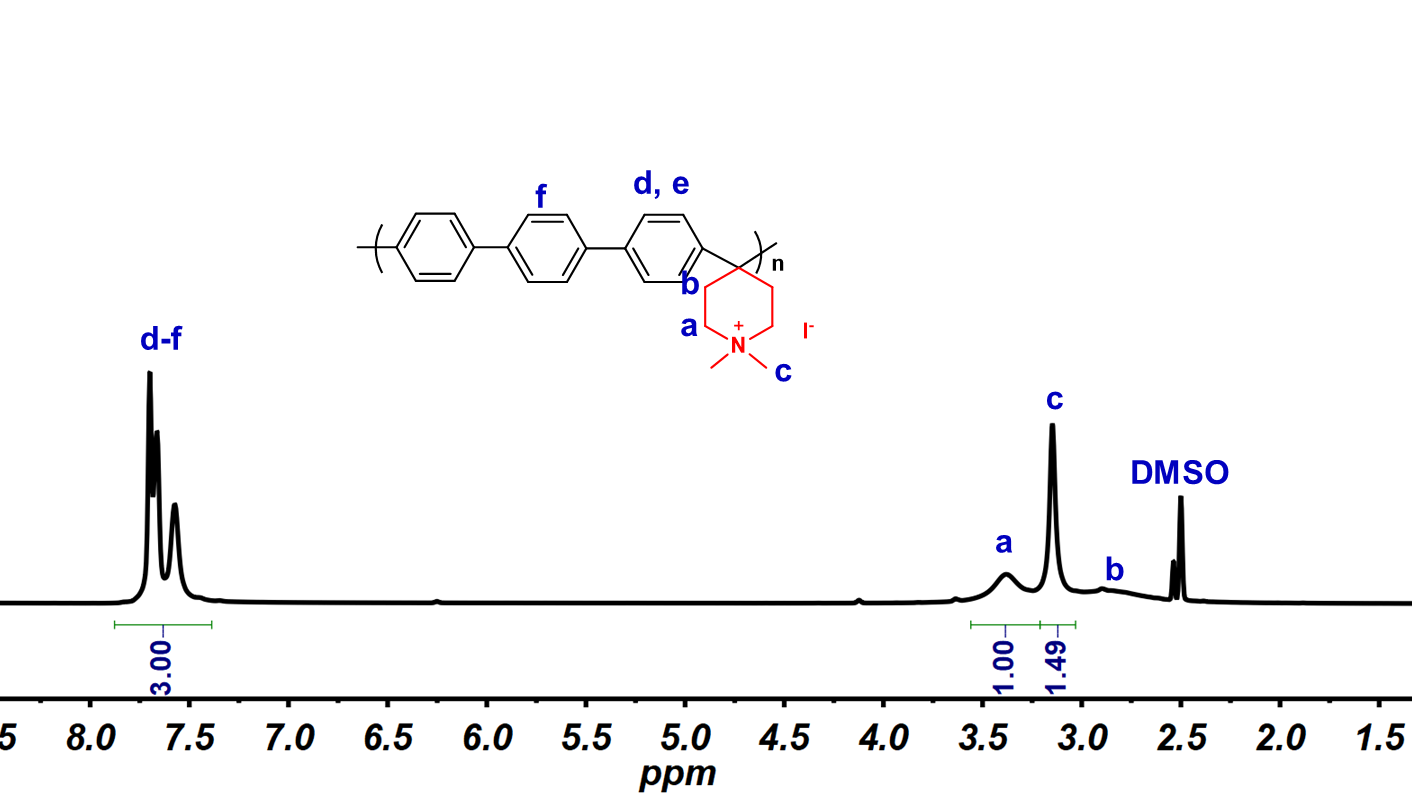


**Supplementary Figure 6.** ^1^H NMR spectrum of PFTP-0 with 10% TFA as a co-solvent in DMSO-d_6_. PFTP-0 is the quaternized product of PFTM-0.


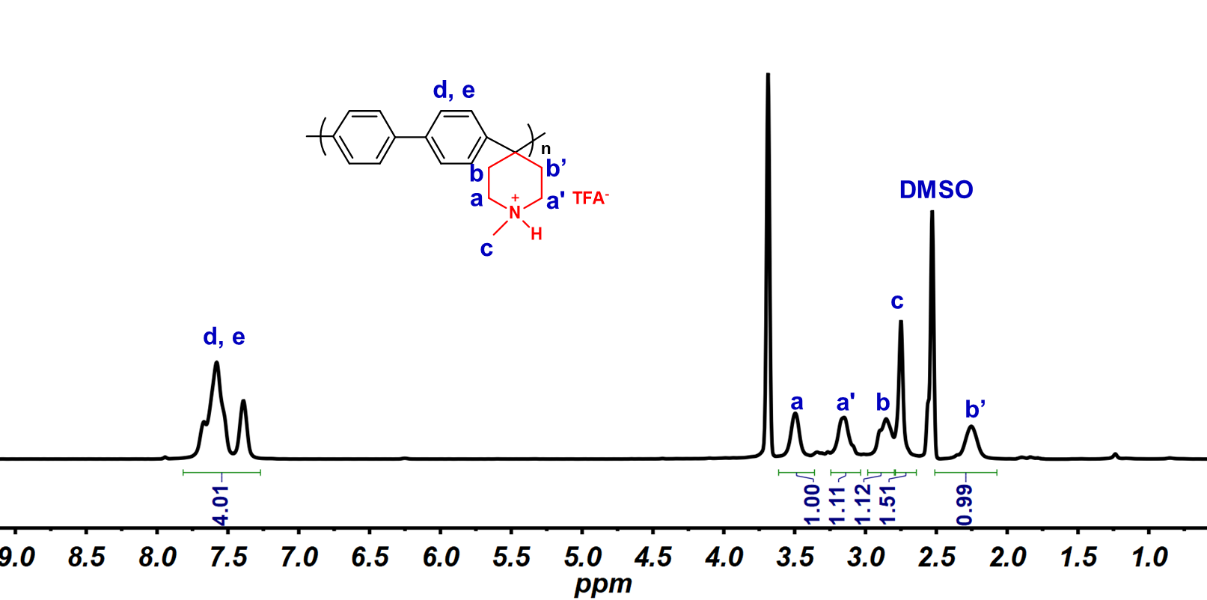


**Supplementary Figure 7.** ^1^H NMR spectrum of PFBM-0 with D_2_O as a co-solvent in DMSO-d_6_. PFBM-0 is the product before quaternization.


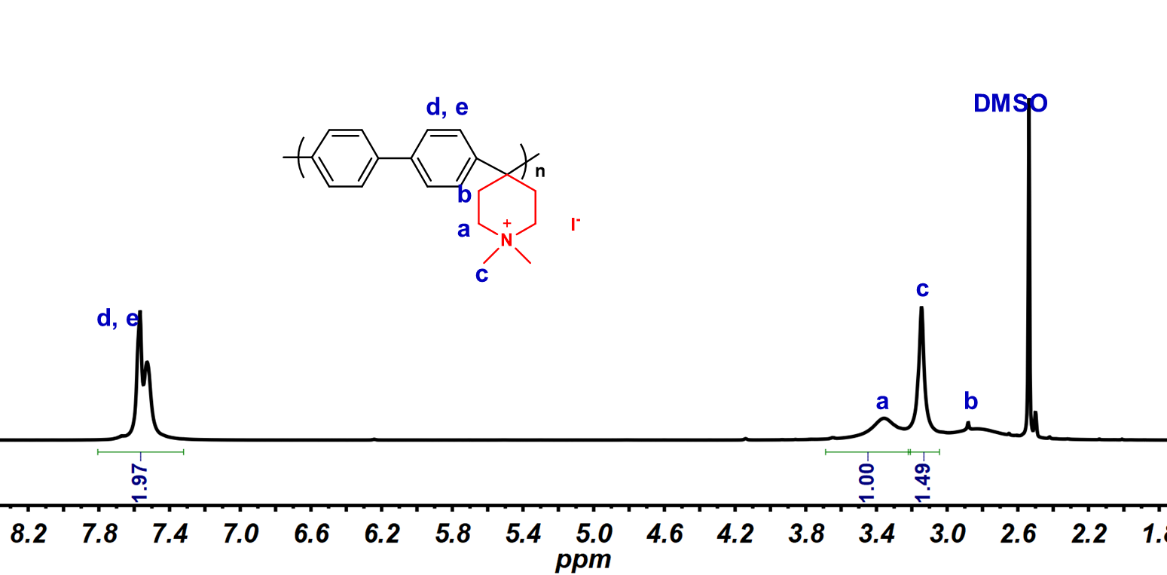


**Supplementary Figure 8.** ^1^H NMR spectrum of PFBP-0 with 10% TFA as a co-solvent in DMSO-d_6_. PFBP-0 is the quaternized product of PFBM-0.


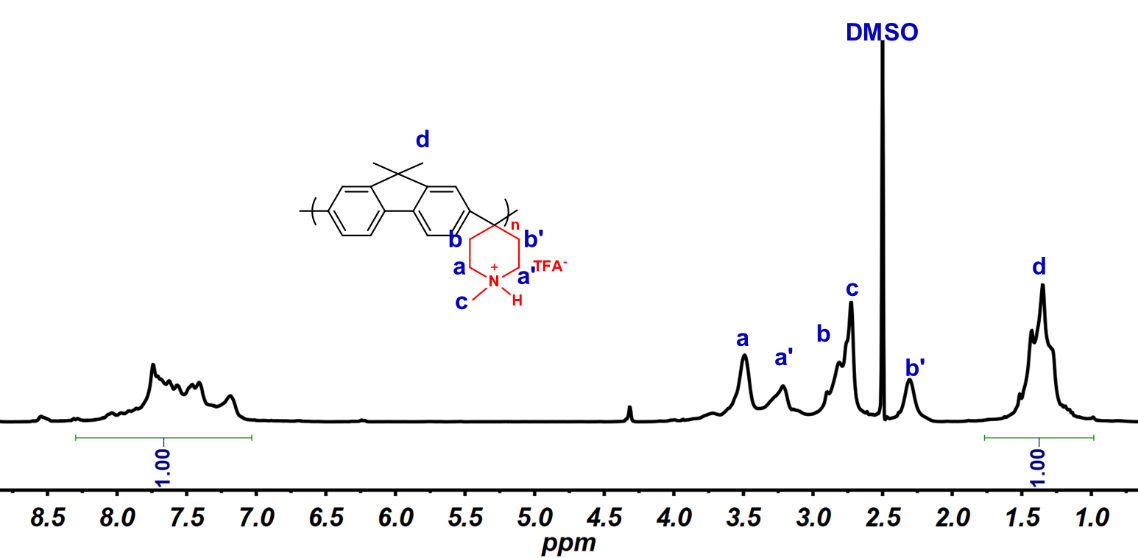


**Supplementary Figure 9.** ^1^H NMR spectrum of PFMN-100 with 10% TFA in DMSO-d_6_. PFMN-100 is the product before quaternization.

**
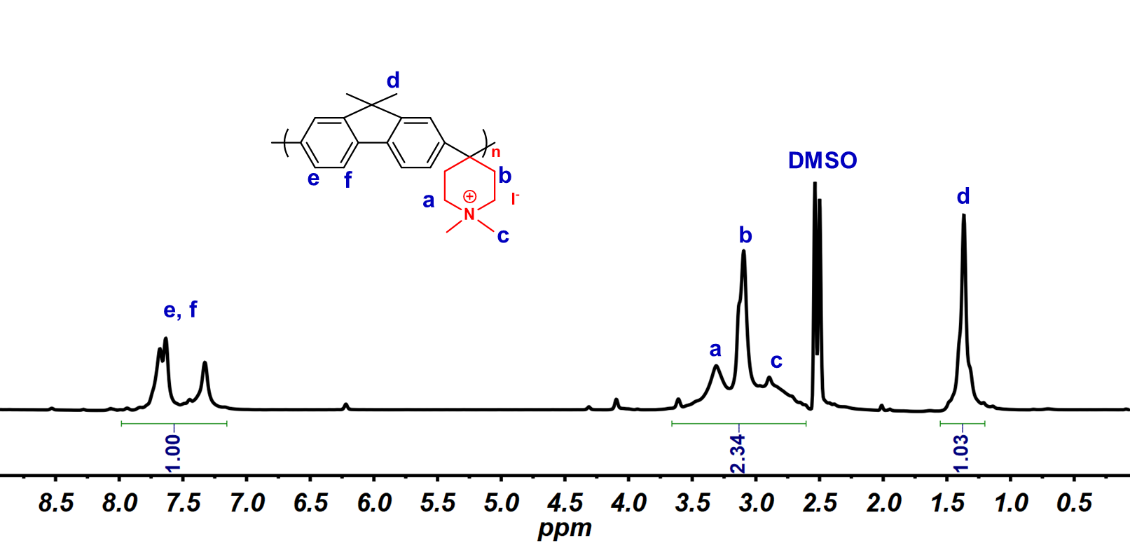
**

**Supplementary Figure 10.** ^1^H NMR spectrum of PFPN-100 with 10% TFA as a co-solvent in DMSO-d_6_. PFPN-100 is the quaternized product of PFMN-100.

**
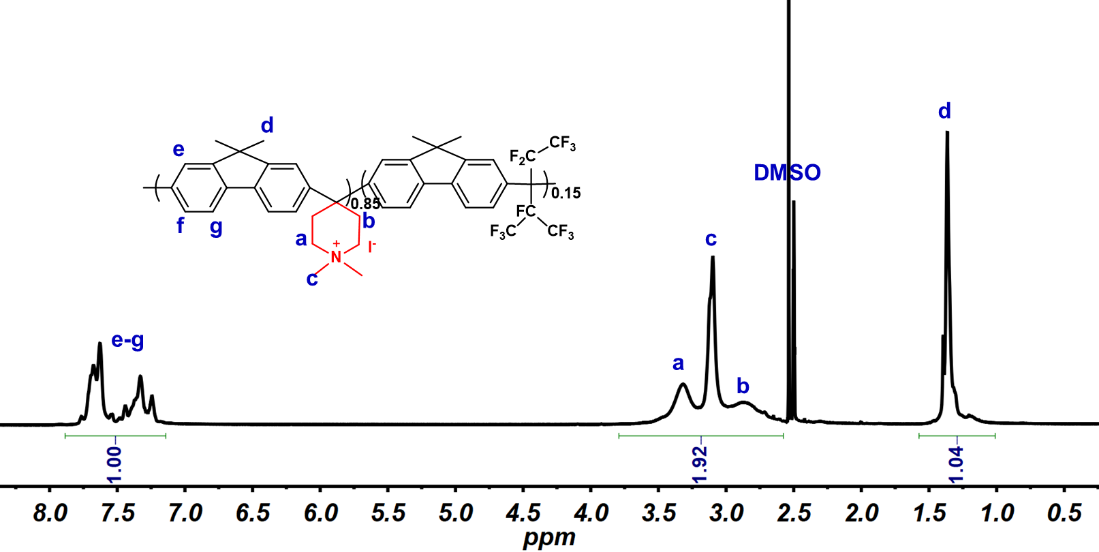
**

**Supplementary Figure 11.** ^1^H NMR spectrum of PFPN-85 with 10% TFA as a co-solvent in DMSO-d_6_. PFPN-85 is the product after quaternization.


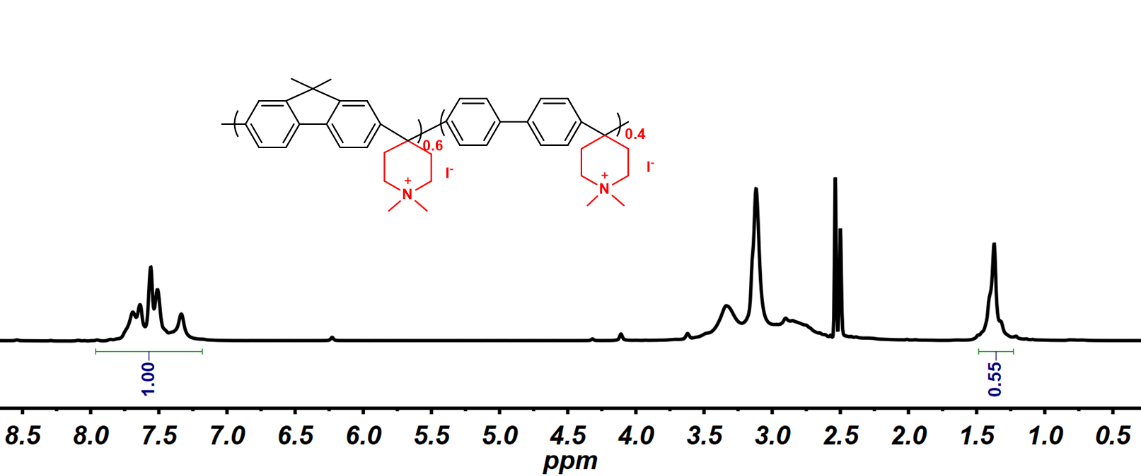


**Supplementary Figure 12.** ^1^H NMR spectrum of PFBP-60 with 10% TFA as a co-solvent in DMSO-d_6_.


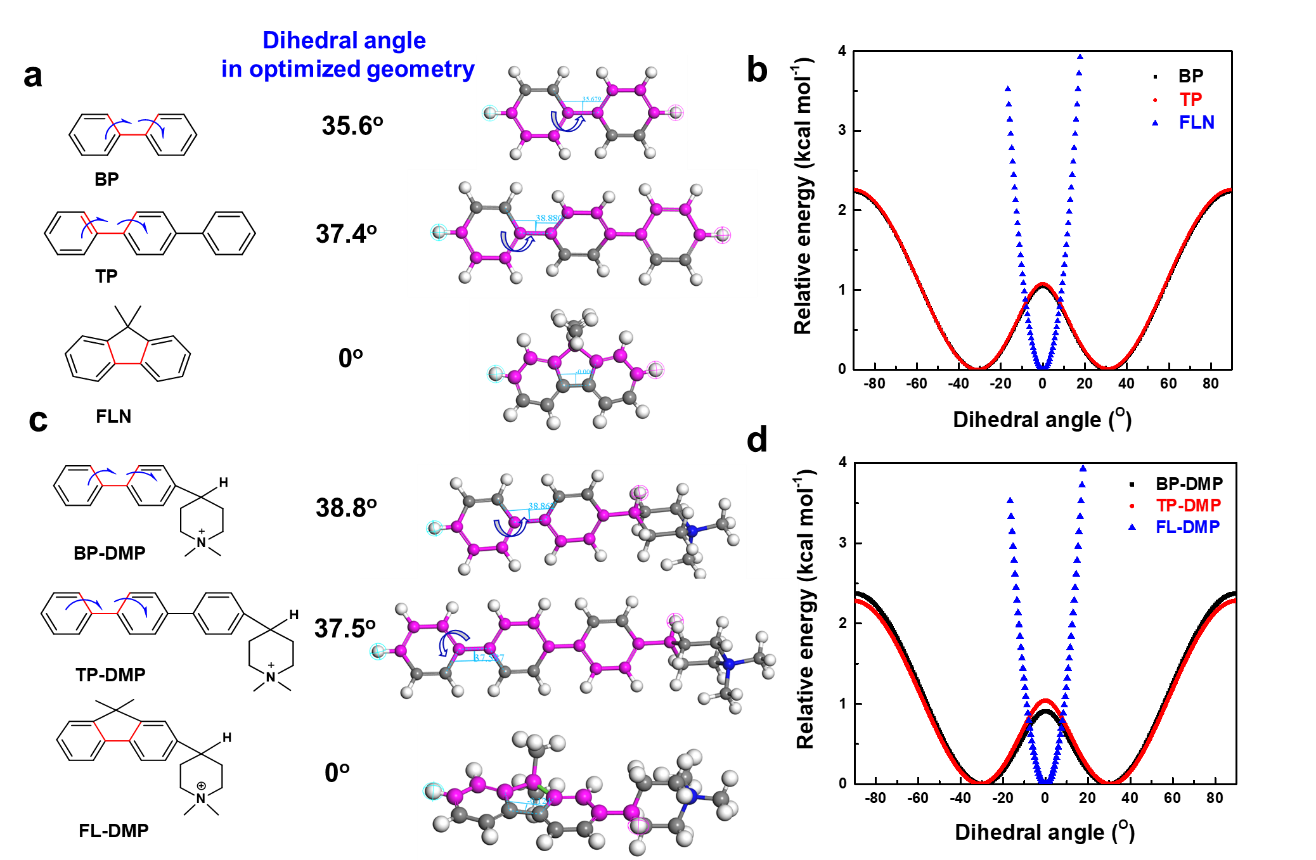


**Supplementary Figure 13. Relative energy variation (kcal mol^-1^) and dihedral angle (⸰) of different molecules. a,** optimized geometry of biphenyl (BP), terphenyl (TP), and 9,9-dimethylfluorene (FLN) represented in ball & stick model, **b,** relative energy variation (kcal mol^-1^) and dihedral angle (⸰) of BP, TP, and FLNs. **c,** optimized geometry of repeat units—biphenyl dimethylpiperidinium (BP-DMP), terphenyl dimethylpiperidinium (TP-DMP), and 9,9-dimethylfluorene dimethylpiperidinium (FL-DMP) displayed in ball & stick model. **d**, relative energy variation (kcal mol^-1^) and dihedral angle (⸰) of BP-DMP, TP-DMP, and FL-DMP. Numbers between figures are dihedral angles of model compounds and repeat units of polymers.

**
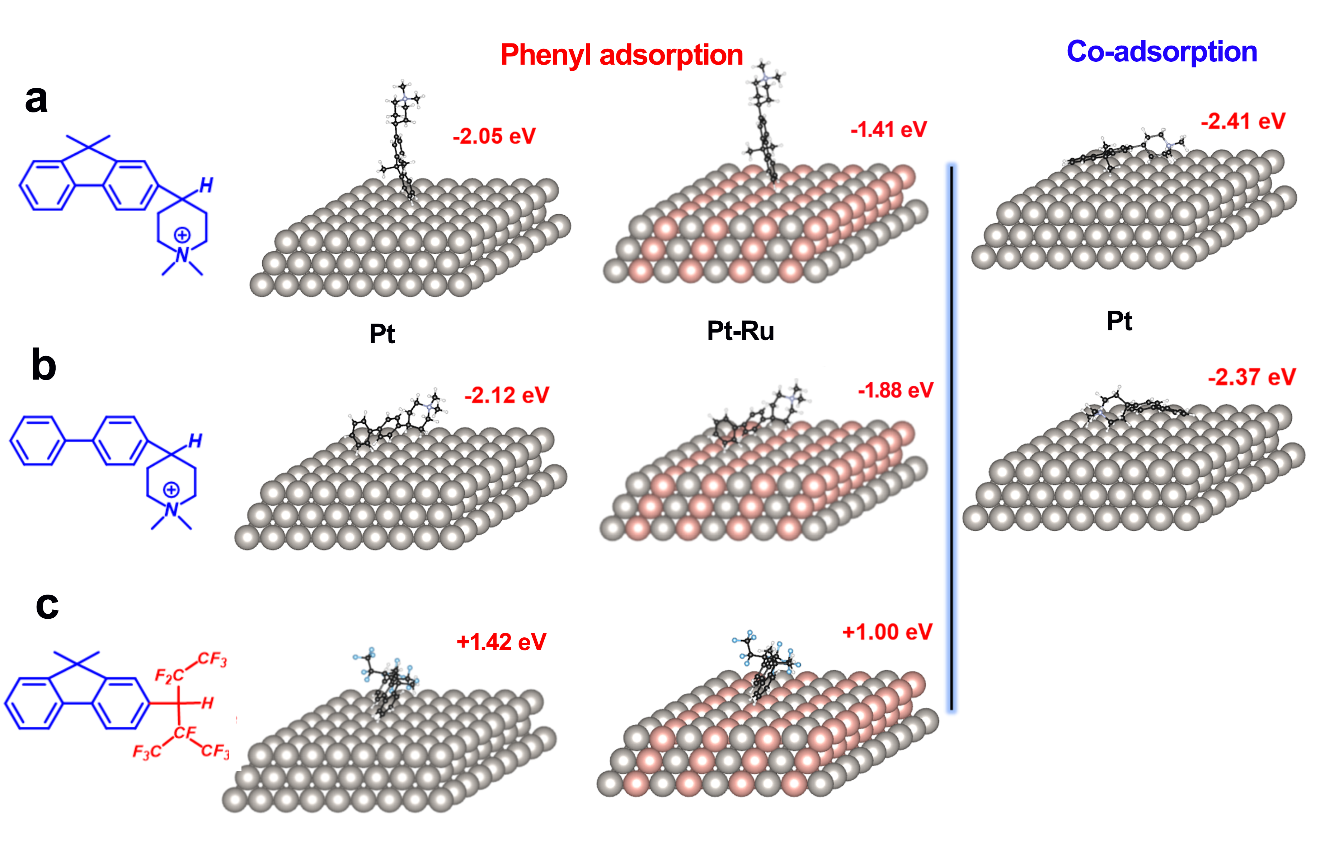
**

**Supplementary Figure 14. Phenyl adsorption and phenyl-ammonium co-adsorption energy (eV) of different molecules with optimized geometries on (111) crystal face of Pt and Pt-Ru by density functional theory** **(DFT) calculations:** **a**, FL-DMP, **b**, BP-DMP, **c,** 9,9-dimethylfluorene nonafluoride (FL-NF). Gray=Pt, pink=Ru, black=C, silver=N, and white=H. The phenyl adsorption energies of BP-DMP and FL-DMP are very close to those reported ^1,2^ by Kim et al.

**
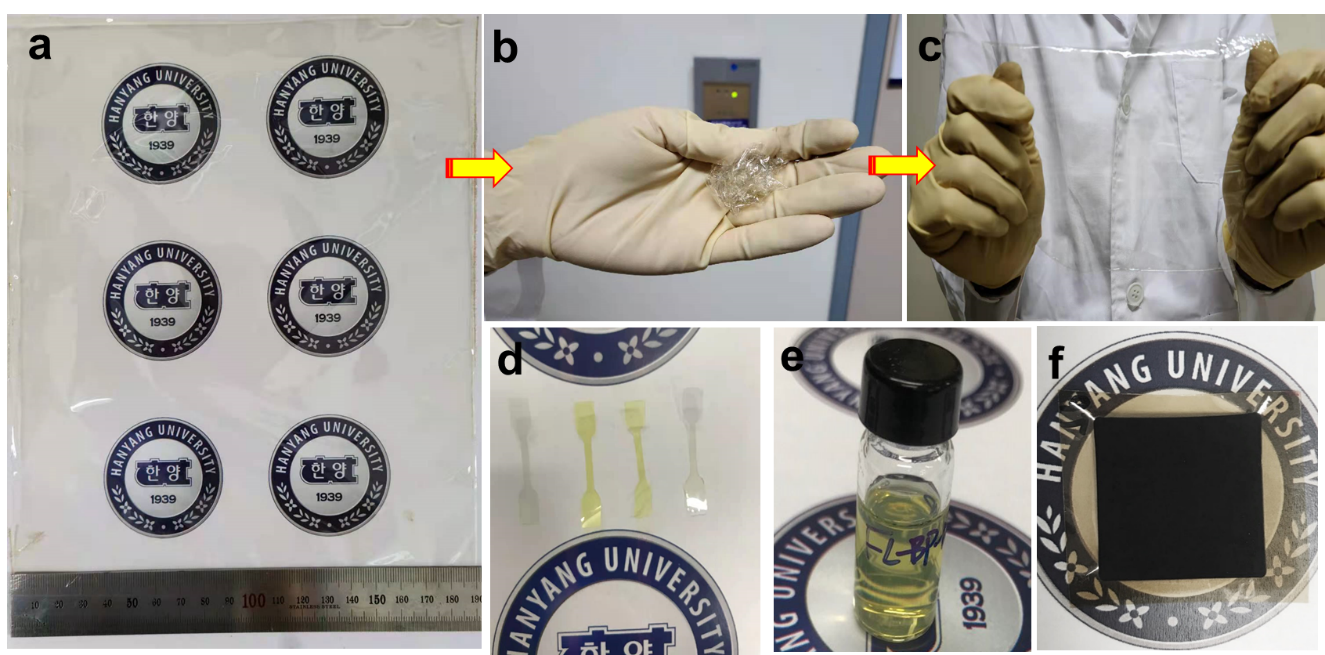
**

**Supplementary Figure 15. Photographs of PFAP membranes and AEIs. a**, PFTP-13 membrane in 19×25 cm. **b,** Folding thin PFTP-13 membrane (9 µm). **c,** PFTP-13 after deforming and stretching **d,** PFBP-0, PFBP-14, and PFTP-13 membranes for mechanical property testing. **e,** PFBP-14 ionomer solution in IPA/DI water for MEA preparation. **f,** Picture of the prepared carbon coated membrane (CCM) with a thin PFTP-13 membrane (20±3µm).


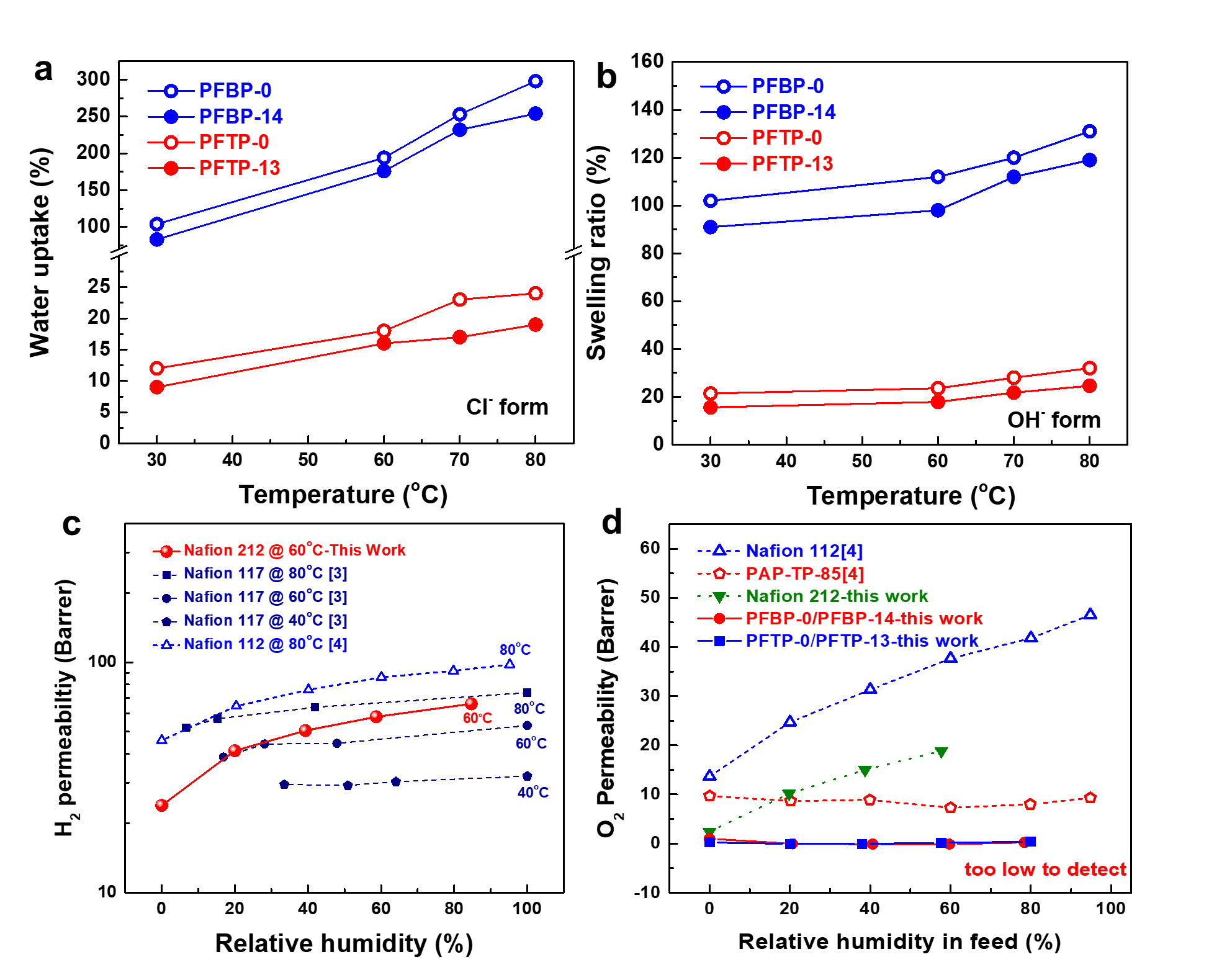


**Supplementary Figure 16.** **a**, water uptake of AEMs in Cl^−^ form and **b,** swelling ratio of AEMs in OH^−^ form. **c,** Comparison of H_2_ permeability of Nafion membranes in this work with reference data^3,4^. **d**, O_2_ permeability of AEMs in this work and reported data^3^.

**
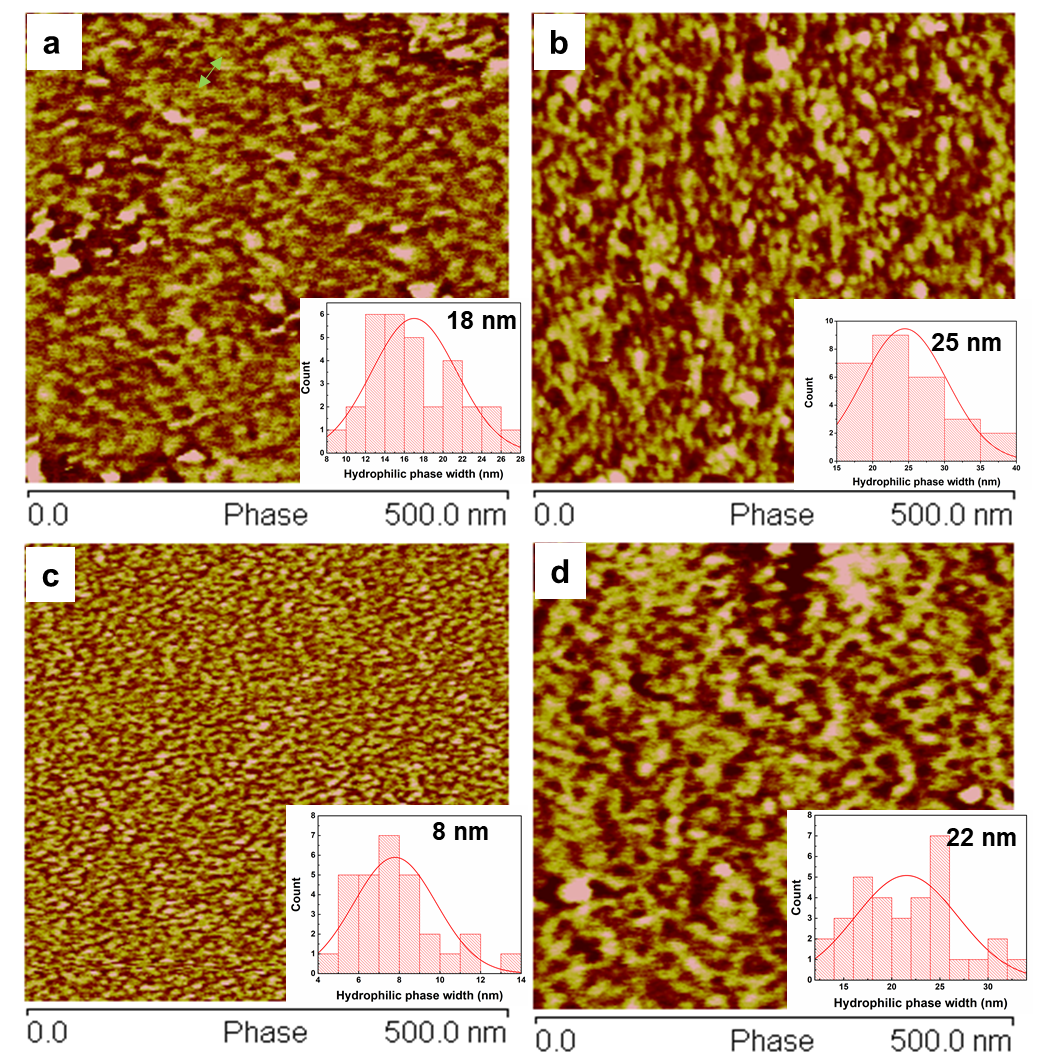
**

**Supplementary Figure 17. Microphase morphology of AEMs. a** PFBP-0, **b**, PFBP-14, **c**, PFTP-0, and **d**, PFTP-13. Black parts in (a) – (d) show hydrophilic regions, while white or yellow parts are regarded as hydrophobic regions. The normal distribution of hydrophilic channel widths in (a) PFBP-0, (b) PFBP-14, (c) PFTP-0 and (d) PFTP-13 are estimated to be around 18 nm, 25 nm, 8 nm and 22 nm, respectively. The hydrophobic widths of AEMs were counted using NanoScope software.


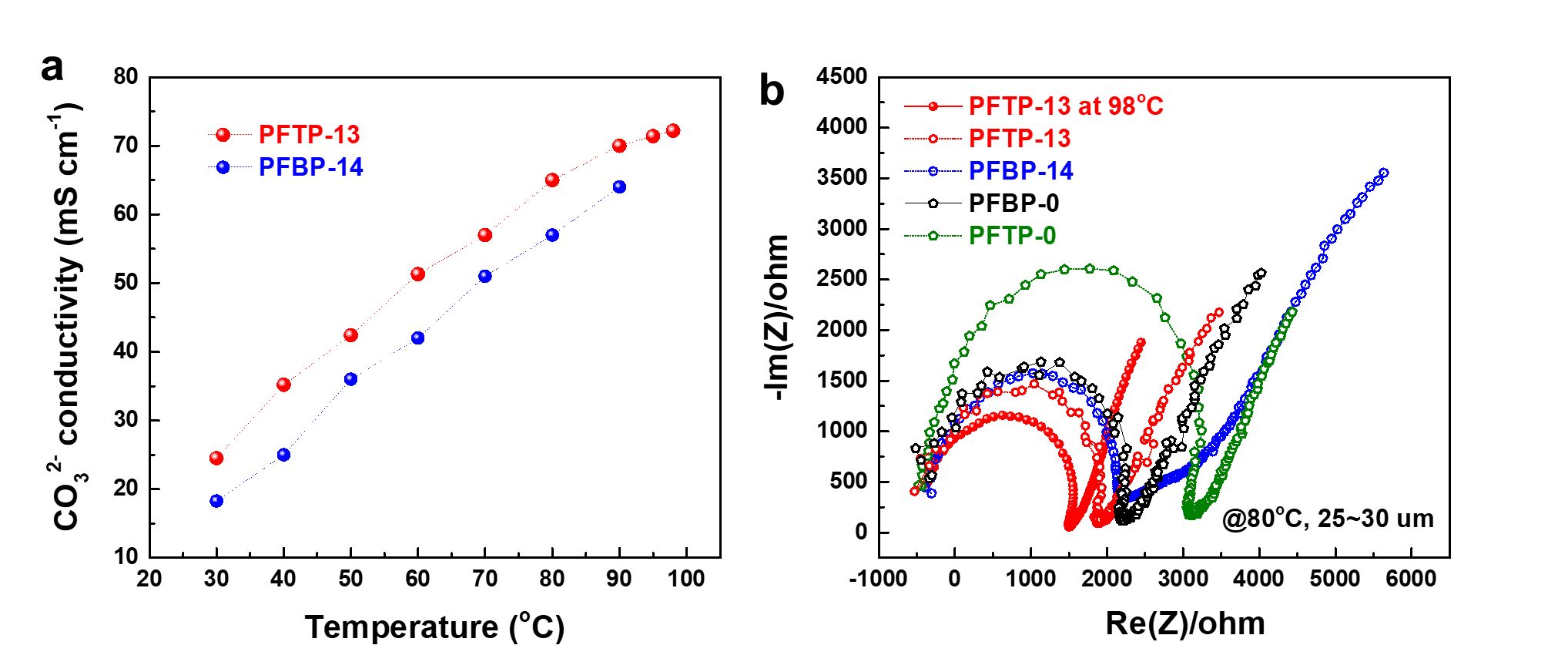


**Supplementary Figure 18. a**, CO_3_^2−^ conductivity of PFTP-13 and PFBP-14 membranes. **b**, Ohmic resistance of PFTP-13, PFBP-14, PFBP-0, and PFTP-0 AEMs at 80 ^o^C in OH^−^ form. Ohmic resistance of PFTP-13 (1470 Ω at 98 ^o^C and 1860 Ω at 80 ^o^C) is lower than PFBP-14 (~2,100 Ω), PFBP-0 (~2,200 Ω), and PFTP-0 (~3000 Ω) in a similar membrane area, which means that the PFTP-13 possesses highest ion-conducting efficiency than PFBP-14.


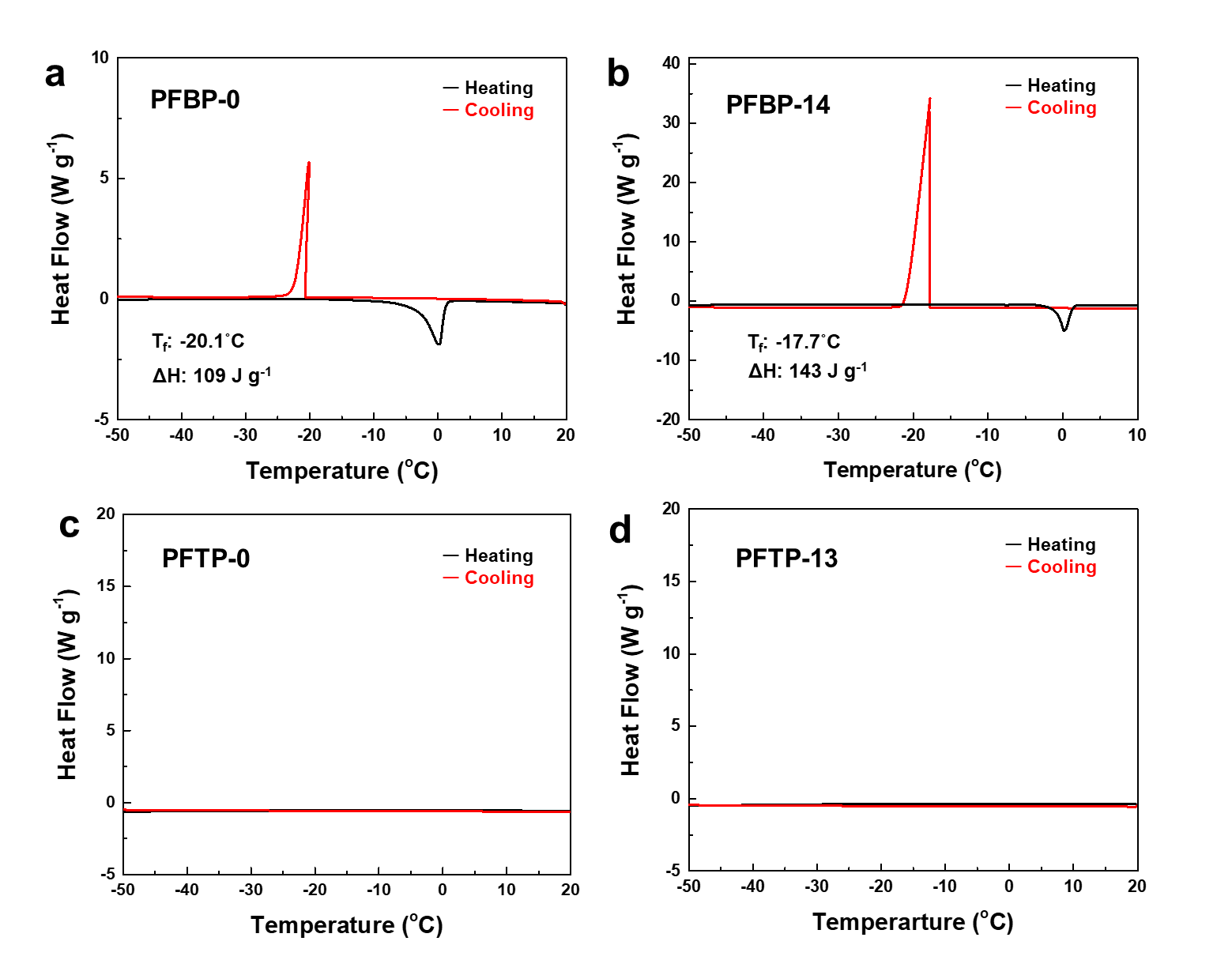


**Supplementary Figure 19. Differential scanning calorimetry (DSC) analysis**. **a**, PFBP-0, **b**, PFBP-14, **c,** PFTP-0 and **d,** PFTP-13 AEMs at OH^−^ form. PFBP (IEC=3.43 mmol g^-1^, *N*_free_=21, *N*_bound_=31) and PFTP (IEC=2.82 mmol g^-1^, *N*_free_=0, *N*_bound_=14) show larger *N*_free_ than PFBP-0 (IEC=3.52 mmol g^-1^, *N*_free_=31, *N*_bound_=24) and PFTP-0 (IEC=2.78 mmol g^-1^, *N*_free_=0, *N*_bound_=17), respectively.

**
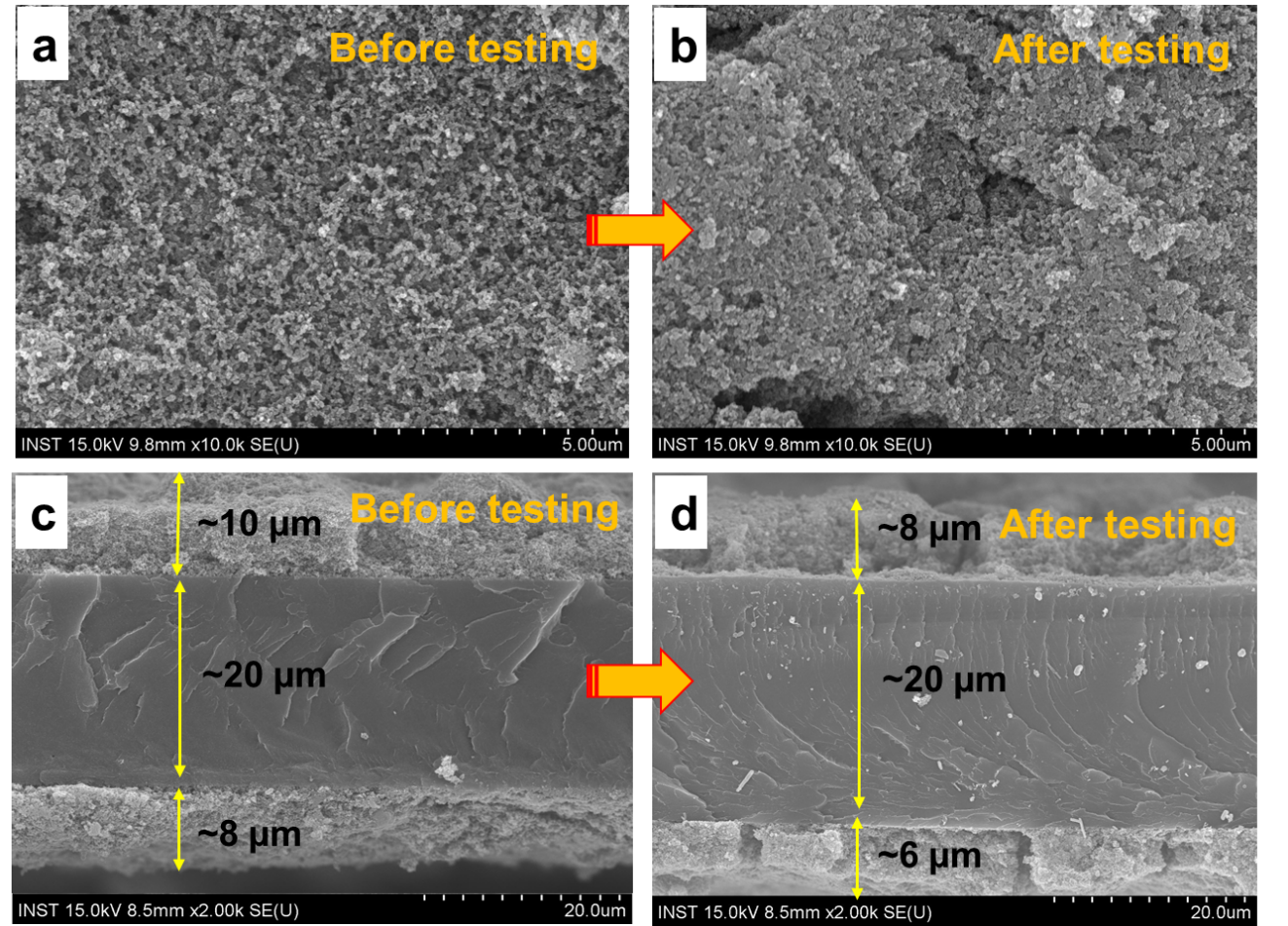
**

**Supplementary Figure 20. SEM image of MEA before and after testing**. **a** and **b**, surface morphology. **c** and **d,** cross-sectional morphology. Before testing, catalyst layer in **a** is porous and loosened, while the catalyst layer became dense and blocky in **b** after testing due to the pressure during cell assembly. The thickness of the catalyst layer and PFTP-13 membrane are about 10 µm and 20 µm from **c** and **d**, respectively.

**
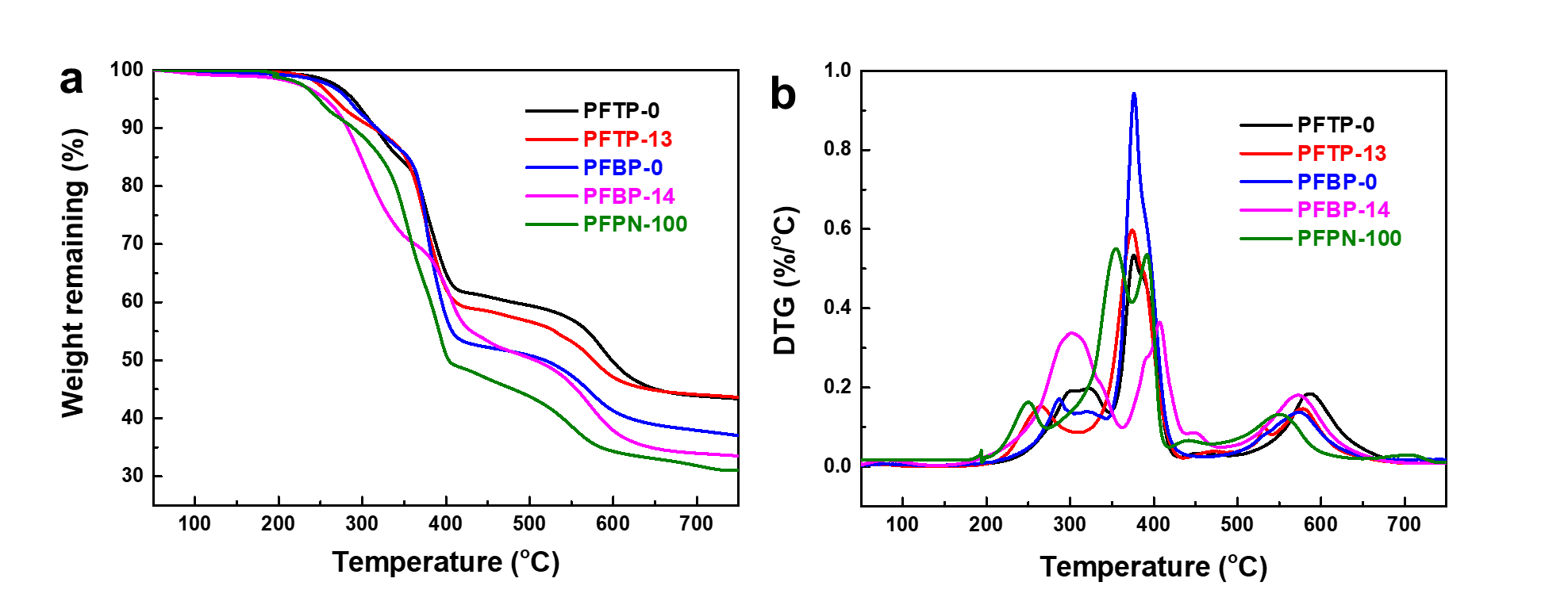
**

**Supplementary Figure 21.** TGA of different AEMs under an N_2_ atmosphere with a temperature range from 30 ^o^C to 800 ^o^C along with a heating speed of 10 ^o^C min^-1^. Every sample was isothermal at 150 ^o^C for 5 min to remove solvents and water before recording data. The first weight stage between 210 to 350 ^o^C is assigned to the decomposition of ammonium groups, and further weight loss over 400 ^o^C is ascribed to the decomposition of the poly(aryl piperidinium) (PAP) backbone.

**
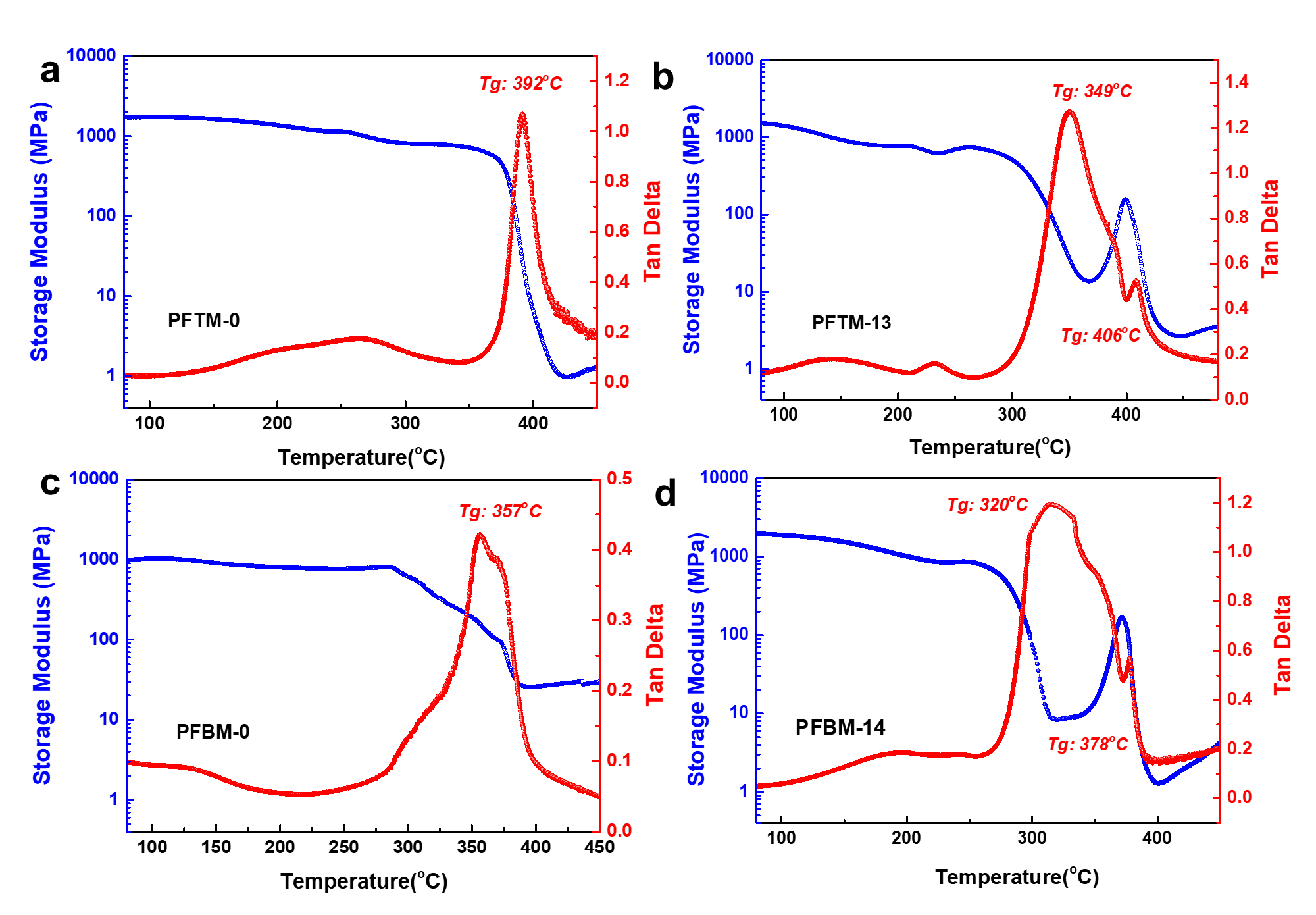
**

**Supplementary Figure 22. Storage modulus and Tan delta**. **a**, PFTM-0, **b**, PFTM-13, **c**, PFBM-0, **d**, PFBM-14 membranes.


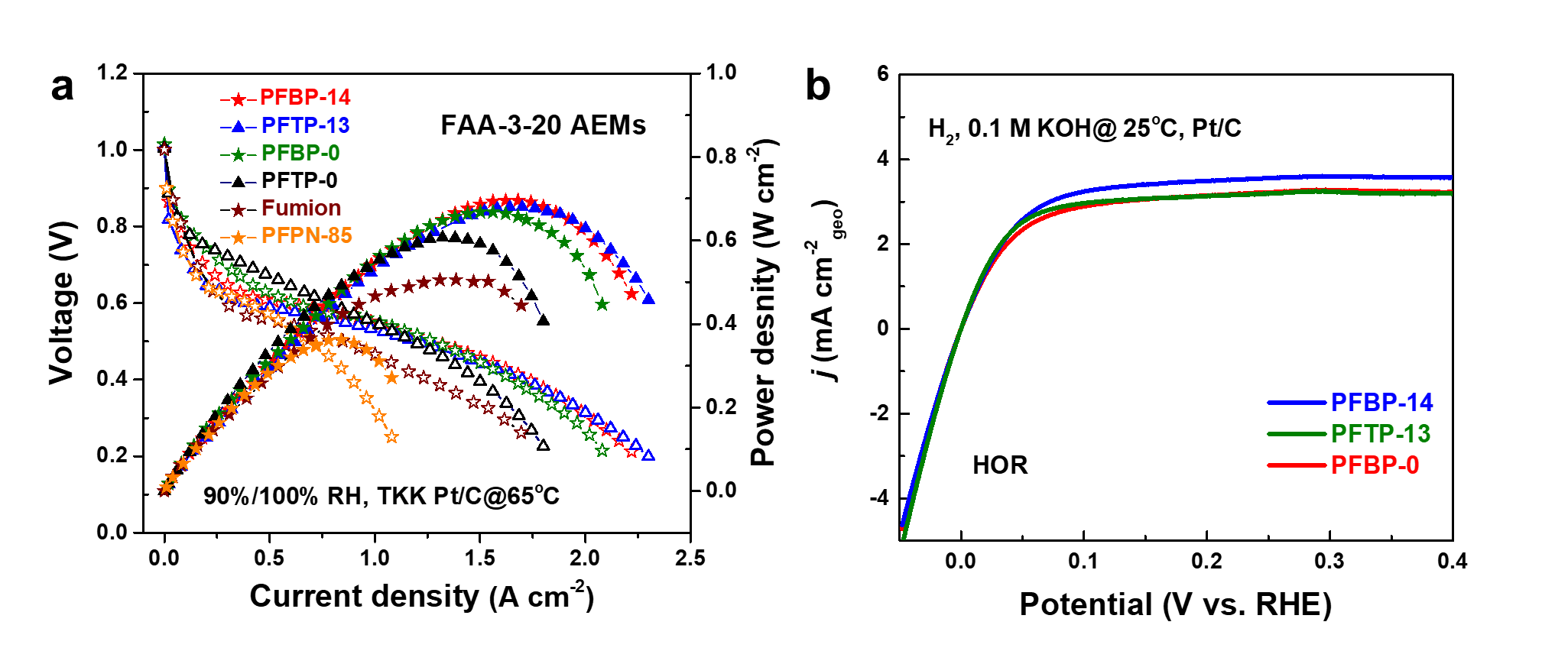


**Supplementary Figure 23. The ionomer effect on AEMFCs and hydrogen oxidation reaction (HOR). a,** H_2_-O_2_, commercial FAA-3-20 membrane, different AEIs, 0.33 mg cm^-2^ loading of A/C TKK Pt/C, at 65 ^o^C, 90%/100% A/C RH, 1,000/1,000 mL min^-1^ A/C flow rate. **b**, HOR voltammograms of Pt/C microelectrodes in contact with four different ionomers. The voltammograms were measured in a saturated H_2_ environment in 0.1 M KOH at 25 ^o^C with a scan rate of 5 mV s^-1^and a rotation speed of 2500 rpm. Basically, four types of AEIs didn’t show significant adsorption effect on HOR performance. FLN-containing AEIs (such as PFBP-14) showed slightly higher HOR current densities of Pt than pristine PFBP-0 or PFTP-13 AEIs due to the lower ionomer adsorption.


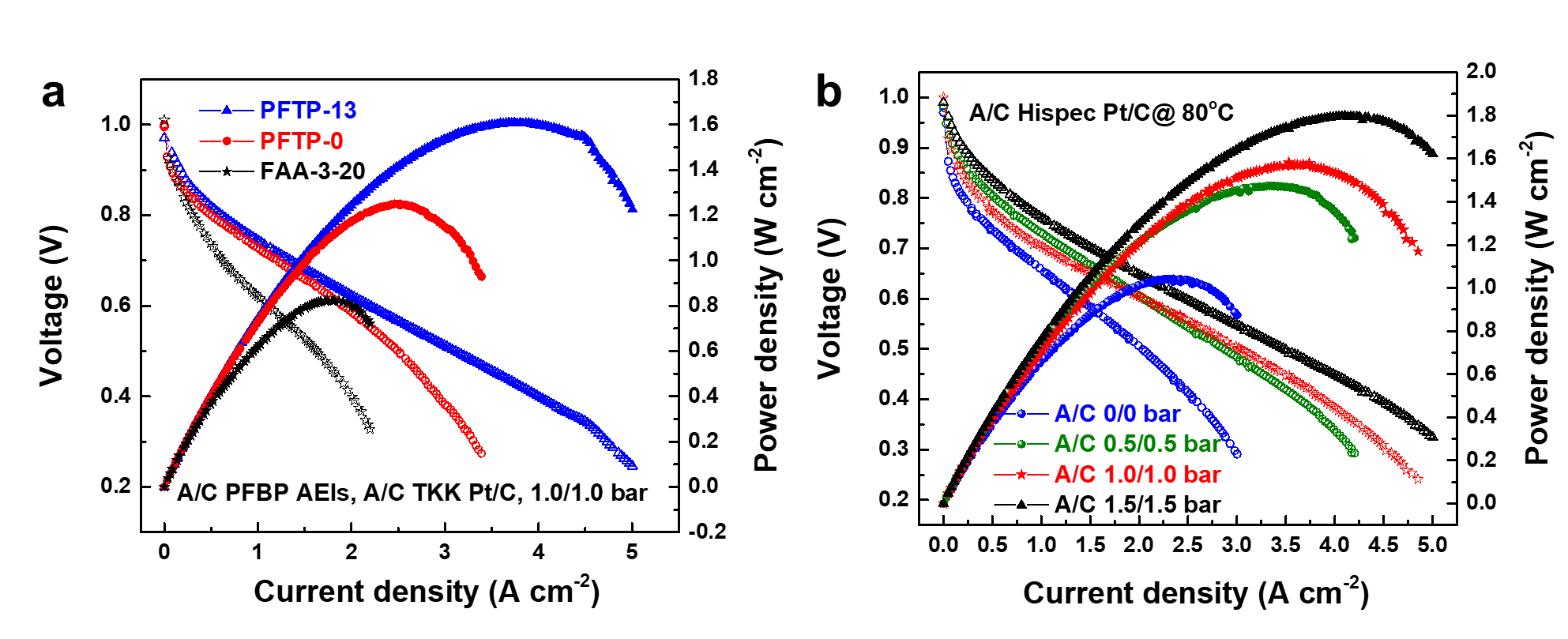


**Supplementary Figure 24.** **a**, comparison of AEMFCs between different AEMs with similar thickness (~25µm) based on A/C PFBP-14 AEIs and A/C TKK Pt/C at 80 ^o^C with 1000/1000 mL min^-1^ H_2_-O_2_ flow rate. **b**, AEMFC performance based on the PFTP-13 membrane and A/C PFBP-14 ionomers with A/C Hispec Pt/C at 80 ^o^C with 1000/1000 mL min^-1^ H_2_-O_2_ flow rate with different back pressures.


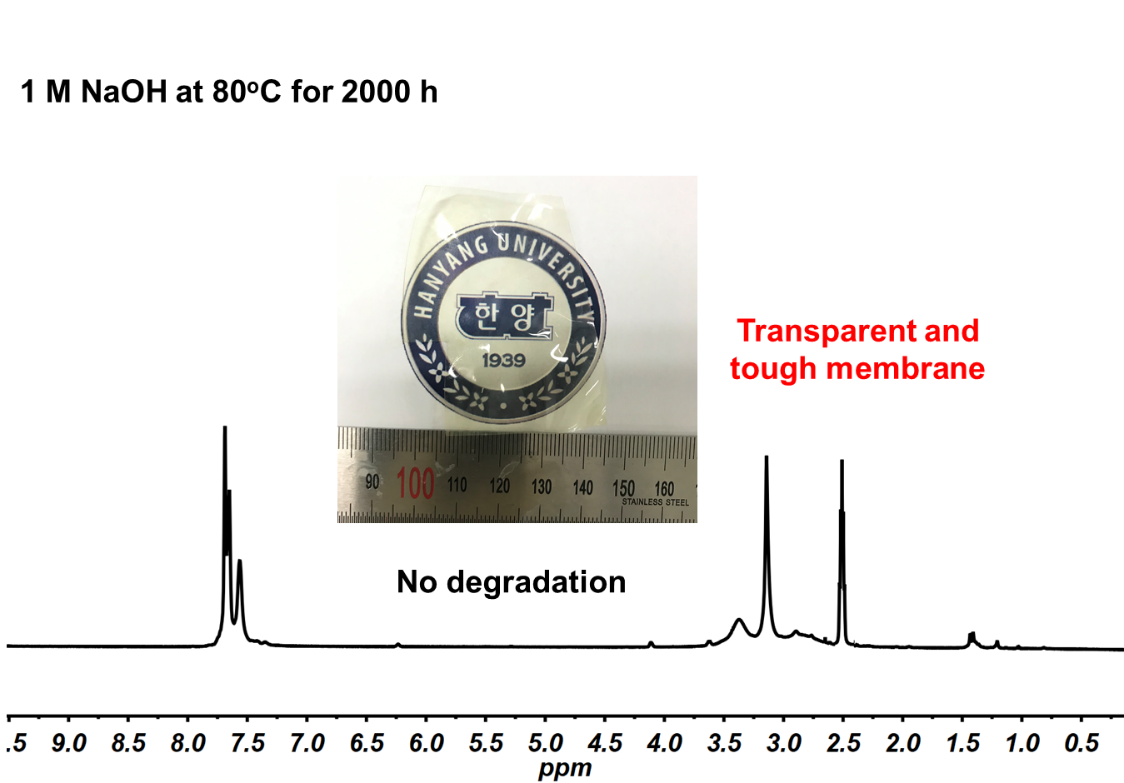


**Supplementary Figure 25.** ^1^HNMR spectrum and picture of PFTP-13 membrane after alkaline treatment in 1 M NaOH at 80 ^o^C for 2,000 h.


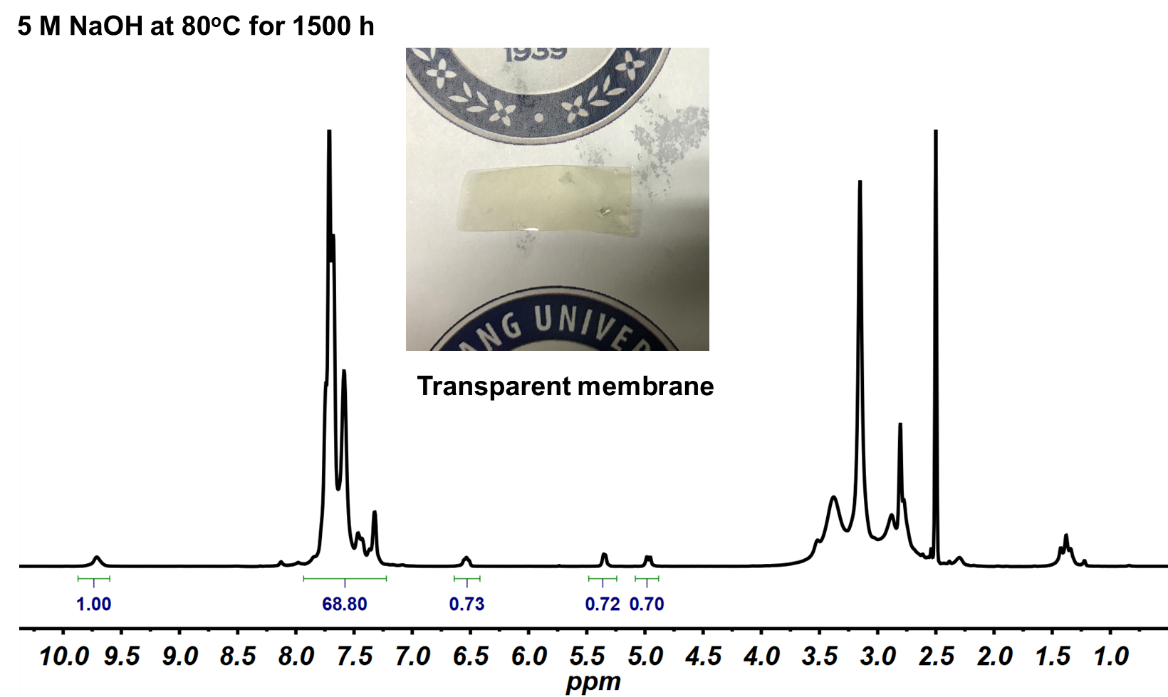


**Supplementary Figure 26.** ^1^HNMR spectrum and picture of PFTP-13 membrane after alkaline treatment in 5 M NaOH at 80 ^o^C for 1,500 h. ~17% degradation of DMP groups was detected.


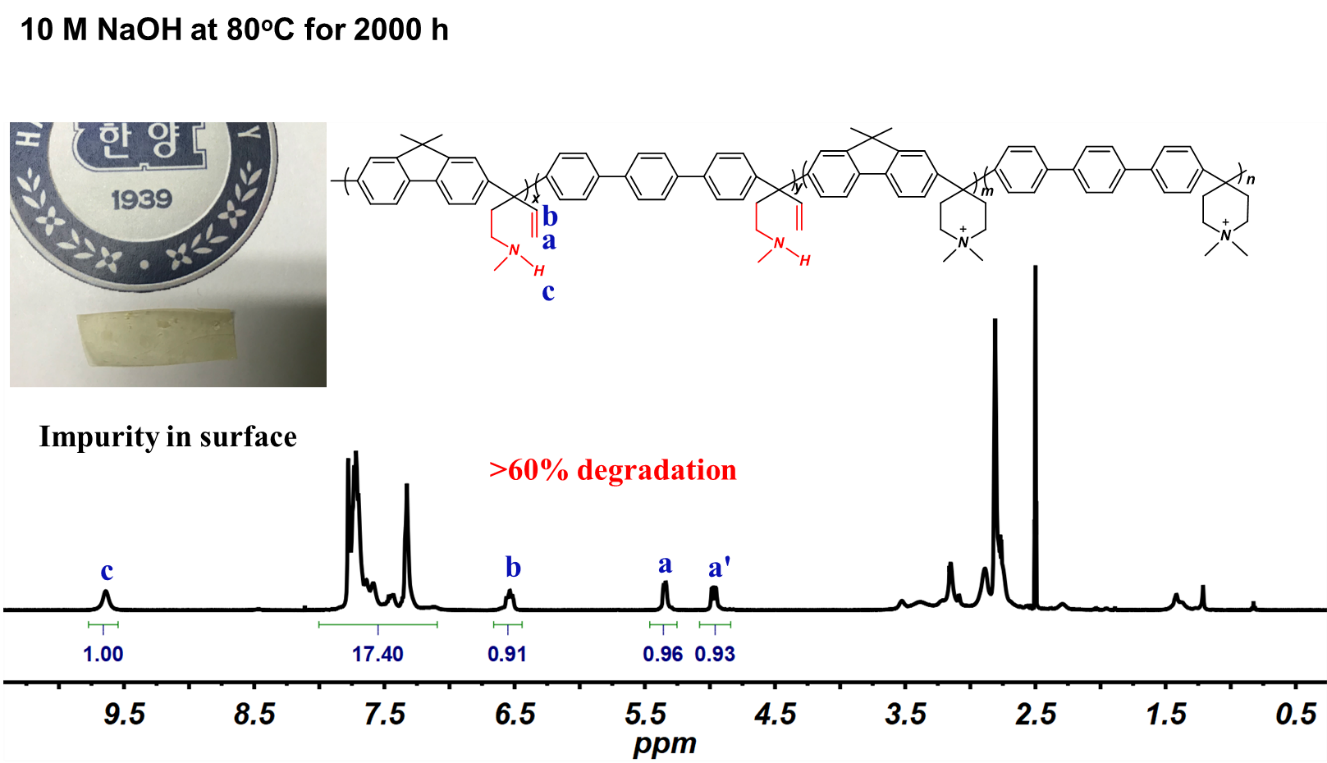


**Supplementary Figure 27.** ^1^HNMR spectrum and picture of PFTP-13 membrane after alkaline treatment in 10 M NaOH at 80 ^o^C for 2,000 h. >60% degradation of DMP groups was detected.


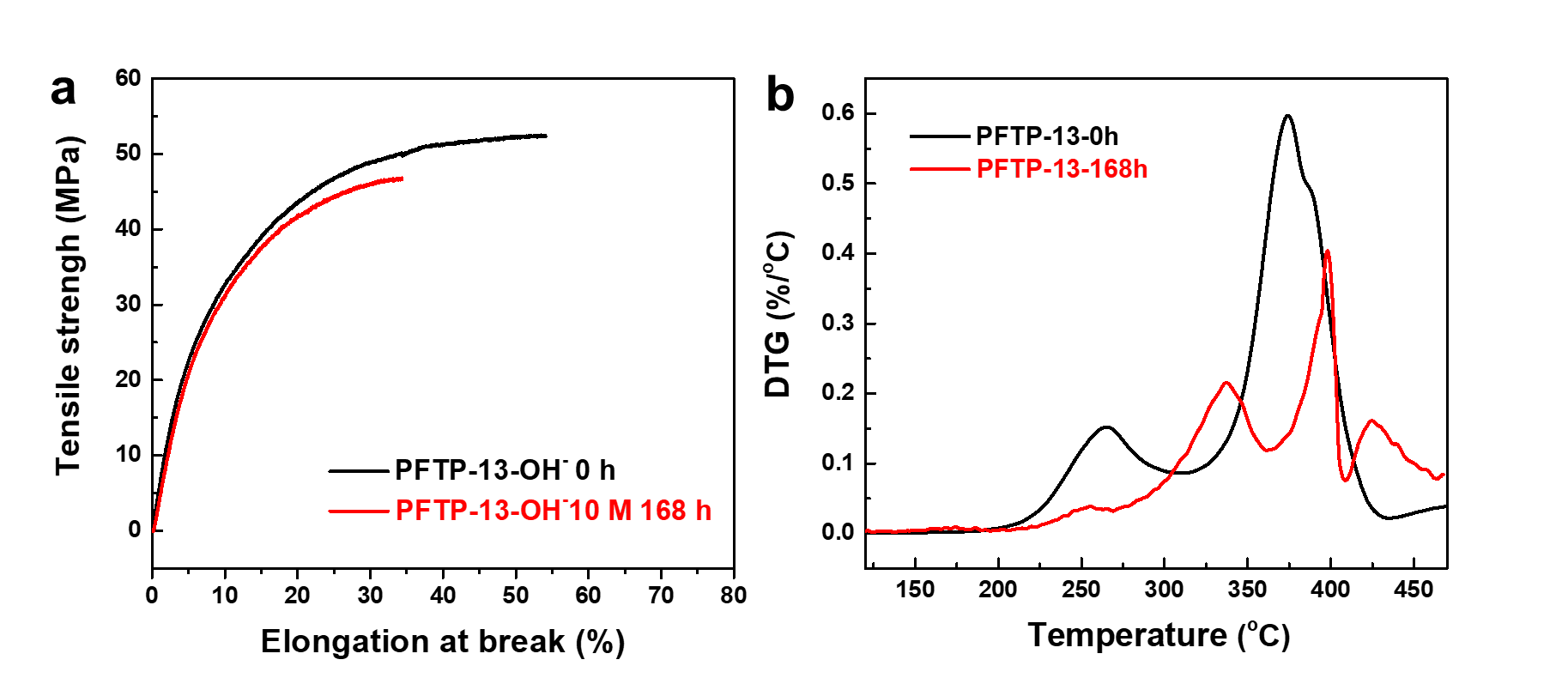


**Supplementary Figure 28.** The variation of **a**, mechanical properties (in OH^−^ form and wet state) and **b**, thermal stability of PFTP-13 in 10 M NaOH at 80 ^o^C for 168 h.





**Supplementary Figure 29.** *In-situ* durability of FAA-3-20-based MEA at 0.2 A cm^−2^ at 60 ^o^C with 200/200 mL min^-1^ flow rate without backpressure. Over 40% voltage decay was found within 40 h.


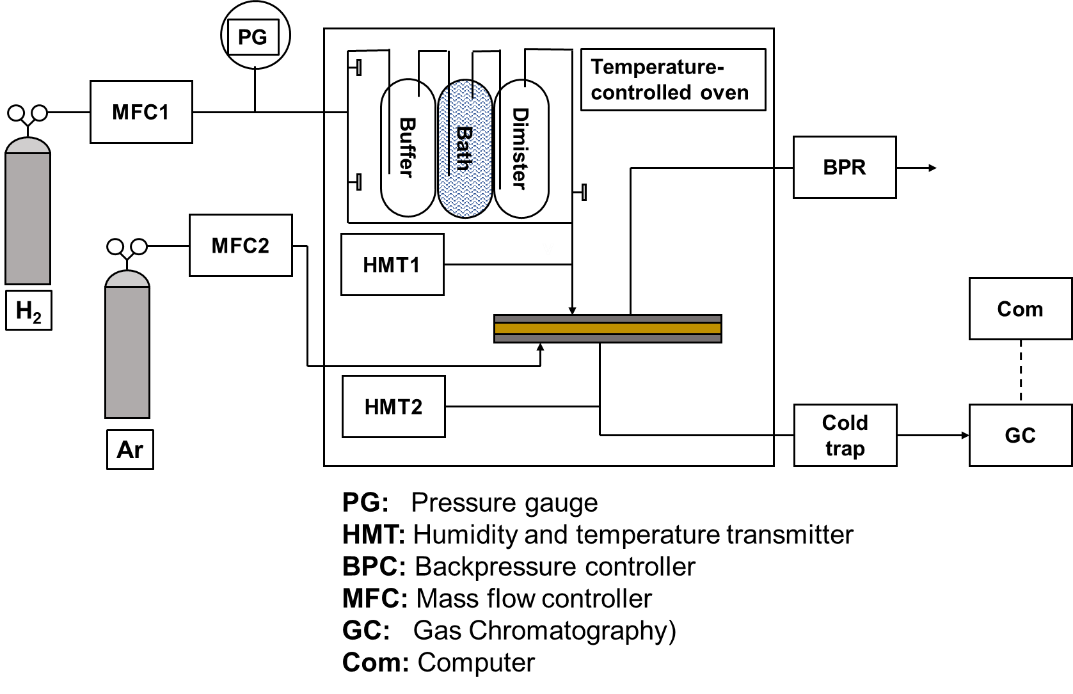


**Supplementary Figure 30.** Schematic diagram of laboratory-made gas and water vapor permeability testing system.

| **Supplementary Table 1.** **Solubility of different polymers in common solvents** | | | | | |
| --- | --- | --- | --- | --- | --- |
|  | DMSO | NMP | DMF | DMAc | 0.1 mL of 5wt% ionomer/DMSO solution in  1.1 mL IPA/DI water (10:1) |
| PFMN-100 | **++** | **++** | **++** | **++** |  |
| PFPN-100 | **+** | **-+** | **+** | **-+** | **+** |
| PFMN-85 | **++** | **++** | **++** | **++** |  |
| PFPN-85 | **+** | **+** | **+** | **-+** | **-+** |
| PFBM-14 | **-** | **+** | **-** | **-** |  |
| PFBM^+^-TFA^−^-14 | **++** | **+** | **++** | **+** |  |
| PFBM-30 | **+** | **+** | **+** | **+** |  |
| PFBM-50 | **+** | **+** | **+** | **+** |  |
| PFBP-14 | **++** | **+** | **++** | **-+** | **++** |
| PFTM-14 | **-** | **-+** | **-** | **-** |  |
| PFTM^+^-TFA^−^-13 | **++** | **++** | **++** | **++** |  |
| PFTP-13 | **++** | **++** | **++** | **++** | **++** |
| PFBM-0 | **-** | **-** | **-** | **-** |  |
| PB M^+^-TFA^−^ | **++** | **+** | **+** | **+** |  |
| PFBP-0 | **++** | **+** | **+** | **+** | **+** |
| PFTM-0 | **-** | **-** | **-** | **-** |  |
| PFTM^+^-TFA^−^-0 | **+** | **+** | **+** | **+** |  |
| PFTP-0 | **+** | **-+** | **-+** | **-** | **+** |
| ++: dissolves well without heating  +: dissolves well with heating  -+: partially dissolved  -: did not dissolve | | | | | |

| **Supplementary Table 2. IEC, WU, SR, hydration number (λ), OH^−^ conductivity, and ŋ of AEPs** | | | | | | | | | | |
| --- | --- | --- | --- | --- | --- | --- | --- | --- | --- | --- |
| **Samples** | IEC (mmol g^-1^) | | | WU (%)^a^ | SR (%)^a^ | λ | *N* _free_ | *N* _bound_ | σ (OH^−^)  (mS cm^-1^) ^a^ | [ŋ] (dL g^-1^)^b^ |
|  | Theo  (OH^−^) | Theo  (I^−^) | NMR  (I^−^) |  |  |  |  |  |  |  |
| PFBP-0 | 3.52 | 2.54 | 2.54 | 350±20 | 107±5 | 55 | 31 | 24 | 58±2 | 5.23 |
| PFPN-100 | 3.09 | 2.31 | 2.31 | 78±12 | − | 14 | − | − | NA | 0.28 |
| PFBP-14 | 3.43 | 2.49 | 2.49 | 300±15 | 94±5 | 52 | 21 | 31 | 63±3 | 2.34 |
| PFPN-85 | 2.86 | 2.25 | 2.25 | 46±12 | − | 9 | − | − | NA | 0.38 |
| PFTP-0 | 2.78 | 2.13 | 2.13 | 55±10 | 24±4 | 17 | 0 | 17 | 48±2 | 4.875 |
| PFTP-13 | 2.82 | 2.16 | 2.16 | 45±5 | 16±3 | 14 | 0 | 14 | 66±4 | 4.08 |
| Fumion | NA | | | 56±5 | 20.25 | NA | − | − | 26.7±2 | − |
| FAA-3-20 | 1.72 | | | 7.0 (Cl^−^ form) | <2.0 (Cl^−^ form) | NA | − | − | 40±2 | − |
| a: at 30 ^o^C in water. b: at 25 ^o^C in DMSO. −: cannot test. NA: not available. *N* *_free_*: number of free water molecules,  *N* *_bound_*: number of bound water molecules. Error bars of WU, SR, and ion conductivity represent that each sample was conducted three time. | | | | | | | | | | |

| **Supplementary Table 3.** **Intrinsic viscosity of ionomers in DMSO at 25 ^o^C.** | | | | | | | | | |
| --- | --- | --- | --- | --- | --- | --- | --- | --- | --- |
| Abbreviation | PFTP-0 | PFTP-13 | PFTP-50 | PFPN-100 | PFPN-85 | PFBP-0 | PFBP-14 | PFBP-30 | PFBP-50 |
| [ŋ] (dL g^-1^) | 4.875 | 4.08 | 0.68 | 0.25 | 0.38 | 5.25 | 2.32 | 1.16 | 0.41 |

| **Supplementary Table 4.** **Water diffusivity of PFBP-0, PFBP-14, PFTP-0, and PFTP-13 AEMs** **in OH^−^ form at different RHs measured at 25 ^o^C.** | | | | |
| --- | --- | --- | --- | --- |
| RH | Water diffusivity (10^-7^ cm s^-1^) | | | |
|  | PFBP-0 | PFBP-14 | PFTP-0 | PFTP-13 |
| 18 % RH | 1.33 | 1.45 | 0.43 | 0.51 |
| 36% RH | 1.43 | 2.08 | 0.67 | 1.13 |
| 54% RH | 3.45 | 3.79 | 0.95 | 1.94 |
| 72% RH | 2.32 | - | 1.36 | 2.53 |
| 90% RH | 2.15 | 2.73 | 1.24 | 1.87 |

| **Supplementary Table 5. Ultimate tensile strength, elongation at break, and E’ (80 ^o^C) of the state-of-the-art AEMs.^6-10^** | | | | | |
| --- | --- | --- | --- | --- | --- |
| Samples | TS (MPa) | EB (%) | YM (MPa) | E’ (MPa) | Ref |
| PFTM-13 | 83.2 | 10.3 | 2152 | 1610 | This work |
| PFTP-13 (I^−^) | 84.6 | 25.6 | 1582 | _ | This work |
| PFTP-13 (OH^−^) | 48.5 | 30.2 | 521 | _ | This work |
| PFTP-0 (I^−^) | 71.1 | 45.7 | 1201 | 1780 | This work |
| PFTP-0 (OH^−^) | 52±5 | 71 | 368 | _ | This work |
| PFBP-14 (I^−^) | 52.9 | 13.6 | 1052 | 2050 | This work |
| PFBP-0 (I^−^) | 46 | 14.8 | 987 | 1050 | This work |
| FAA-3-20 | 47 | 12.5 | 876 | _ | This work |
| PTFE-reinforced PNB | NA | NA | NA | 553 | [5] |
| PAP-TP-x (HCO_3_^−^) | 67 | 117 | NA | NA | [3] |
| QAPPT  (OH^−^) | 34.8 ± 2.6 | 39.5 ± 7.9 | NA | NA | [10] |
| BTMA-HDPE  (Cl^−^) | 35 | 283 | NA | NA | [6] |
| BTMA-LDPE (Cl^−^) | 23 | 69 | NA | NA | [6] |
| PAImBB (Cl^−^) | 65.2 ± 5.3 | 20.4 ± 1.8 | 1095 ± 54 | NA | [7] |
| PAImEE (Cl^−^) | 64.0 ± 11.6 | 28.7 ± 8.0 | 1075 ± 212 | NA | [7] |
| PX75-T50 | 264 | 1.8 | 172 | NA | [8] |
| PX10-T50 | 144 | 2.5 | 117 | NA | [8] |

| **Supplementary Table 6. Summary of fuel cell performance in this work along with number of repetitions in this work and for typical AEMFCs^1,11-20^**. | | | | | | |
| --- | --- | --- | --- | --- | --- | --- |
| AEIs | AEMs | PPD (W cm^-2^) | Catalyst types | | Repeat times | Ref |
|  |  |  | Pt/C anode | Pt-Ru/C anode |  |  |
| Fumion | FAA-3-20 | 0.3 to 0.5  (with BP) | 0.33 mg cm^-2^ |  | >10 | This work |
|  | FAA-3-50 | 0.3 to 0.4  (with BP) | 0.33 mg cm^-2^ |  | >5 | This work |
|  | PFTP-13 | 0.4 to 0.65 (with BP) | 0.33 mg cm^-2^ |  | 3 | This work |
|  | PFTP-0 | 0.4 to 0.5  (with BP) | 0.33 mg cm^-2^ |  | 2 | This work |
| PFPN-85 | FAA-3-20 | 0.15 to 0.35 (with BP) | 0.33 mg cm^-2^ |  | >5 | This work |
|  | PFTP-13 | 0.25 to 0.65 (with BP) | 0.33 mg cm^-2^ |  | >5 | This work |
| PFPN-100 | FAA-3-20 | 0.20 to 0.40 (with BP) | 0.33 mg cm^-2^ |  | 3 | This work |
|  | PFTP-13 | 0.300 to 0.68  (with BP) | 0.33 mg cm^-2^ |  | >5 | This work |
| PFTP-13 | FAA-3-20 | 0.59 to 0.70 (with BP) | 0.33 mg cm^-2^ |  | >6 | This work |
|  | PFTP-13 | 0.9 to 1.3  (with BP) | 0.33 mg cm^-2^ |  | >5 | This work |
| PFBP-14 | FAA-3-20 | 0.7 to 0.82  (with BP) | 0.33 mg cm^-2^ |  | >5 | This work |
|  | PFTP-13 | 0.75 to 1.25 (no BP) | 0.33 mg cm^-2^ |  | >30 | This work |
|  |  | 1.3 to 1.8 (with BP) |  |  |  |  |
|  | PFTP-13 | 1.4 to 1.67  (no BP) |  | 0.33 mg cm^-2^ or 0.42 mg cm^-2^ | >20 | This work |
|  |  | 2.05 to 2.34 (with BP) |  |  |  |  |
|  | PFTP-0 | 0.6 to 1.0  (no BP) | 0.33 mg cm^-2^ |  | ~5 | This work |
|  |  | 0.98 to 1.4 (BP) |  |  |  |  |
| PFBP-0 | PFTP-13 | 0.6 to 0.9  (no BP) | 0.33 mg cm^-2^ |  | >5 | This work |
|  |  | 1.0 to 1.5 (BP) |  |  |  |  |
| PFTP-0 | PFTP-13 | 0.8 to 1.1  (with BP) | 0.33 mg cm^-2^ |  | 3 | This work |
| PFBP-0 | PFTP-0 | 0.55 to 0.7  (no BP) | 0.33 mg cm^-2^ |  | 3 | This work |
| PPO-ASU | PFTP-13 | 0.25 to 0.35 | 0.33 mg cm^-2^ |  | 2 | This work |
| QAPPT | QAPPT | 2.0 |  | 0.4 mg cm^-2^ | NG | [10] |
| PAP-BP-100 | PAP-TP-85 | 1.89 |  | 0.7 mg cm^-2^ | NG | [9] |
| BTMA-ETFE | GT82-15/PTFE | 3.0 to 3.45 |  | 0.7 mg cm^-2^ | NG | [5] |
| BTMA-ETFE | BTMA-HDPE | 2.55 |  | 0.7 mg cm^-2^ | NG | [6] |
| BTMA-ETFE | BTMA-LDPE | 2.05 |  | 0.7 mg cm^-2^ | NG | [6] |
| BTMA-ETFE | BTMA-ETFE | 1.9 |  | 0.72 mg cm^-2^ | NG | [11] |
| FLN-55 | TPN | 1.45 |  | 0.75 mg cm^-2^ | NG | [1] |
| TEA-o-BPN | TPN | >1.5 |  | 0.75 mg cm^-2^ | NG | [12] |
| BPN | TPN | 1.2 |  | 0.75 mg cm^-2^ | NG | [12] |
| BTMA-ETFE | Gen 2 PFAEM | 1.4 |  | 0.75 mg cm^-2^ | NG | [11] |
| BTMA-ETFE | M20C9N6NC5N | 0.94 |  | 0.6 mg cm^-2^ | NG | [13] |
| Crosslinked CBQPPO | TMImPPO | 1.37 |  | 0.5 mg cm^-2^ | NG | [14] |
| aQAPS-S_14_ | aQAPS-S_8_ | 1.0 |  | 0.4 mg cm^-2^ | NG | [15] |
| BTMA-ETFE | PAImBB | 0.25 |  | 0.5 mg cm^-2^ | NG | [7] |
| BTMA-SEBS or FLN-55 | SEBS series | 0.3 to 0.72 |  | 0.3 to 0.75 mg cm^-2^ | NG | [16,17] |
| PBI series | PBI series | <0.4 |  |  | NG | [18] |
| Other PPO series | Other PPO series | 0.05 to 0.4 |  |  | NG | [19,20] |
| PEEK series | PEEK series | Poor performance |  |  | NG |  |
| Commercial AEIs | FAA series and Tokuyama series | ~0.50 |  |  | NG | This work and [3] |

**Supplementary Discussion**

**Fluorene segment calculation**. As shown in Supplementary Figs. 2-3, the fluorene segment ratio (*x*) in fluorene-based copolymers can be calculated by the equation: $\frac{d}{a}=\frac{6x}{2(x+y)}=\frac{6x}{2}$ , where *d* represents six methyl protons in fluorene segments, and *a* represents two protons in the piperidinium ring. Therefore, *x_1_* was calculated to be 0.13 in PFTM-13, and *x_2_* was calculated to be 0.14 in PFBM-14.

**Torsional rotation calculation.** As shown in Supplementary Figs 13a-b, three representative compounds—BP-DMP, TP-DMP, and FL-DMP were used for torsional rotation calculation. FLN-based units have much smaller dihedral angle (D) in the optimized geometry along with much higher rotation energy barrier over the same rotation dihedral angles than biphenyl and terphenyl-based units. High rotation energy barrier implies that FLN-based units are hard to conduct spatial rotation that signifies the high rigidity of FLN groups, which contributes to improving the dimensional stability and water vapor permeability of PFAP copolymers.

**DFT calculation.** As shown in Supplementary Fig. 14, BP-DMP, FL-DMP and FL-NF—were used to investigate the phenyl adsorption and phenyl-ammonium co-adsorption energies on Pt (111) and Pt-Ru (111) using DFT calculations. FLN groups possessed lower phenyl adsorption energy (-1.41 eV and -2.05 eV) than BP (-1.88eV and -2.12eV) on the (111) lattice plane of Pt-Ru and Pt, respectively, due to the hindrance of dimethyl groups that restrict the interactions between the π-system of FLN groups and the catalytic surface. However, the co-adsorption energy of DMP and aromatic groups on catalysts has not been investigated. Supplementary Figure 14 shows that the co-adsorption energies of FL-DMP and BP-DMP are significantly higher than the phenyl adsorption energy, implying that DMP groups also have a strong adsorption on Pt and Pt-Ru. Notably, the phenyl adsorption energies of BP-DMP and FL-DMP are very close to (but slightly higher than) those reported ^1,2^ by Kim *et al.* on aryl groups due to the addition of DMP groups in the present study. Unfortunately, the adsorption energy of FL-BP-DMP molecules cannot be calculated by the DFT method due to the excessively large molecular information. The detailed co-adsorption effect of ionomers on catalysts will be further studied in our future work.

**Morphology analysis**. Supplementary Figures 17a-d exhibit AFM images of PFBP-0, PFBP-14, PFTP-0, PFTP-13 membranes in a water swollen state. The bright region belongs to hydrophobic phases that are constructed by the rigid polymer backbone, while the dark region represents hydrophilic phases that are aggregated by piperidinium and water. Specifically, the normal distribution of hydrophilic channel widths in (a) PFBP-0, (b) PFBP-14, (c) PFTP-0 and (d) PFTP-13 AEMs are 18 nm, 25 nm, 8 nm and 22 nm, respectively. Compared to PFTP-0 AEMs, PFBP-0 AEMs possess much larger hydrophilic channel widths due to the larger IEC and hydration number (λ). On the other hand, PFAP AEMs (PFBP-14 and PFTP-13) exhibit distinct and larger micro-phase separated morphologies and channels than those of PAP AEMs (PFBP-0 and PFTP-0) at similar values of λ (PFBP-0 *vs*. PFBP-14 and PFTP-0 *vs*. PFTP-13). It is reasonable that much larger water channels in PFAP (PFBP and PFTP) over PAP (PFBP-0 and PFTP-0) should have improved the water vapor permeability as well as ion conductivity. Although the λ of PFTP-13 (λ=14) is much lower than PFBP-14 (λ=52) and PFBP-0 (λ=55), the PFTP-13 AEMs still display continuous and larger hydrophilic phase widths compared to PFBP-14 and PFBP-0 AEMs, respectively, suggesting that the ion conducting capacity through the PFTP-13 AEMs with low water content (or λ=14) should have been the most efficient one among the PAPs studied in this work.

**Free water and bound water in AEMs.** Supplementary Figure 19 shows that swollen membranes have two types of water, namely bound water and free water^5^. Bound water is defined as water molecules that bound tightly with ionic species in the polymers, which is considered to be beneficial to ion conductivity^5^. Free water is defined as water molecules that surround the bound water. Frozen free water molecules in AEPs melt at around 0 ^o^C upon heating, whereas they refreeze upon cooling. Detailed calculations related to free water and bound water are provided in the method section in the main text. As can be seen in Supplementary Table 2, PFBP-14 and PFBP-0 exhibit much more free water (*N*_free_) and bound water (*N*_bound_) contents than those of PFTP-13 and PFTP-0 due to their large λ values. The PFBP-0 with the higher free water (IEC=3.52 mmol g^-1^, *N*_free_=31, *N*_bound_=24) exhibits a lower ion conductivity compared to PFBP-14 (IEC=3.43 mmol g^-1^, *N*_free_=21, *N*_bound_=31), while PFTP-13 with a lower IEC (IEC=2.82 mmol g^-1^, *N*_free_=0, *N*_bound_=14) displays much higher ion conductivity than PFBP-14 and PFBP-0 AEMs, implying that excessive *N*_free_ may not enhance ion conductivity^5^. On the other hand, PFTP-0 (IEC=2.78 mmol g^-1^, *N*_free_=0, *N*_bound_=17) exhibits the lowest ion conductivity among these AEMs due to their small hydrophilic channel widths (8 nm, Supplementary Fig. 17) and low IEC values.

**DMA analysis.** Supplementary Figure 22 shows the glass-transition temperature (*T_g_*) and storage modulus (E’) of the representative polymers. PFTM-0 (392 ^o^C) and PFBP-0 (357 ^o^C) homopolymers exhibit a single *T_g_*, whereas PFAM copolymers such as PFTM-13 (349 ^o^C, 406 ^o^C) and PFBM-14 (320 ^o^C, 378 ^o^C) exhibit two *T_g_*s, indicating that PFAM copolymers have two different polymer segments with excellent micro-phase separated morphologies of PFAP membranes as shown in Supplementary Fig. 17. On the other hand, these PFAM copolymers exhibited a higher E’ (over 1,500 MPa at 80 ^o^C) than PFTM-0 and PFBP-0 homopolymers, indicating that FLN segments increased the rigidity and mechanical strength of PFAP copolymers. Supplementary Table 4 lists the mechanical properties and E’ of the state-of-the-art AEMs in current research. Compared to PTFE-reinforced crosslinked poly(norbornene) (PNB) membranes (66.8 to 553 MPa)^5^, PFAM copolymers display much higher E’ and tensile strength, indicating that these PFAPs possess high thermomechanical properties.

**Fuel cell performance.** Supplementary Figure 24a reveals that PFTP-13 AEM exhibits higher PPD and limited current density compared to PFTP-0 and commercial FAA-3-20 membranes due to the higher ion conductivity and mechanical properties. On the other hand, the effect of back pressure on AEMFCs was investigated based on the PFTP-13 membrane and PFBP-14 A/C AEIs. Supplementary Figure 24b implies that a small amount of back pressure shows a significant effect on the power density of AEMFCs. Based on Hispec Pt/C, the PPDs of AEMFCs increase from 1.08 W cm^-2^ to 1.80 W cm^-2^ at 80 ^o^C after gradually increasing A/C back pressure from 0 bar to 1.5 bar. Specifically, high back pressure increases the reactant fuel gas pressure which results in higher electrode reaction rates. Moreover, high back pressure also contributes to improving the water permeability of AEMs or AEIs, which decreases the mass transport resistance.

**Supplementary References**

1 Maurya, S. *et al.* Rational design of polyaromatic ionomers for alkaline membrane fuel cells with >1 W cm^−2^ power density. *Energy & Environmental Science* **11**, 3283-3291 (2018).

2 Matanovic, I. *et al.* Adsorption of Polyaromatic Backbone Impacts the Performance of Anion Exchange Membrane Fuel Cells. *Chemistry of Materials* **31**, 4195-4204 (2019).

3 K. BROKA, P. E. Oxygen and hydrogen permeation properties and water uptake of Nafion®117 membrane and recast film for PEM fuel cell. *Journal of applied electrochemistry* **27**, 117-123 (1997).

4 Wang, J. *et al.* Poly(aryl piperidinium) membranes and ionomers for hydroxide exchange membrane fuel cells. *Nature Energy* **4**, 392-398 (2019).

5 (1) Huang, G. *et al.* Composite Poly(norbornene) Anion Conducting Membranes for Achieving Durability, Water Management and High Power (3.4 W/cm^2^) in Hydrogen/Oxygen Alkaline Fuel Cells. *Journal of The Electrochemical Society* **166**, F637-F644 (2019). (2) Mandal, M. *et al.* The Importance of Water Transport in High Conductivity and High-Power Alkaline Fuel Cells. *Journal of The Electrochemical Society* **167** (2019).

6 Wang, L., Peng, X., Mustain, W. E. & Varcoe, J. R. Radiation-grafted anion-exchange membranes: the switch from low- to high-density polyethylene leads to remarkably enhanced fuel cell performance. *Energy & Environmental Science* **12**, 1575-1579 (2019).

7 Fan, J. *et al.* Poly(bis-arylimidazoliums) possessing high hydroxide ion exchange capacity and high alkaline stability. *Nat Commun* **10**, 2306 (2019).

8 Kim, Y. *et al.* Ionic Highways from Covalent Assembly in Highly Conducting and Stable Anion Exchange Membrane Fuel Cells. *J Am Chem Soc* **141**, 18152-18159 (2019).

9 Wang, T. *et al.* High-Performance Hydroxide Exchange Membrane Fuel Cells through Optimization of Relative Humidity, Backpressure and Catalyst Selection. *Journal of The Electrochemical Society* **166**, F3305-F3310 (2019).

10 Li, Q. *et al.* The Comparability of Pt to Pt-Ru in Catalyzing the Hydrogen Oxidation Reaction for Alkaline Polymer Electrolyte Fuel Cells Operated at 80 degrees C. *Angew Chem Int Ed Engl* **58**, 1442-1446 (2019).

11 Omasta, T. J. *et al.* Beyond catalysis and membranes: visualizing and solving the challenge of electrode water accumulation and flooding in AEMFCs. *Energy & Environmental Science* **11**, 551-558 (2018).

12 Park, E. J. *et al.* How does a small structural change of anode ionomer make a big difference in alkaline membrane fuel cell performance? *Journal of Materials Chemistry A* **7**, 25040-25046 (2019).

13 Zhu, L. *et al.* Poly(olefin)-Based Anion Exchange Membranes Prepared Using Ziegler–Natta Polymerization. *Macromolecules* **52**, 4030-4041 (2019).

14 Liang, X. *et al.* Ionomer Cross-Linking Immobilization of Catalyst Nanoparticles for High Performance Alkaline Membrane Fuel Cells. *Chemistry of Materials* **31**, 7812-7820 (2019).

15 Wang, Y. *et al.* Pt–Ru catalyzed hydrogen oxidation in alkaline media: oxophilic effect or electronic effect? *Energy & Environmental Science* **8**, 177-181 (2015).

16 Gao, X. *et al.* Enhanced water transport in AEMs based on poly(styrene–ethylene–butylene–styrene) triblock copolymer for high fuel cell performance. *Polymer Chemistry* **10**, 1894-1903 (2019).

17 Jeon, J. Y. *et al.* Synthesis of Aromatic Anion Exchange Membranes by Friedel–Crafts Bromoalkylation and Cross-Linking of Polystyrene Block Copolymers. *Macromolecules* **52**, 2139-2147 (2019).

18 Wright, A. G. *et al.* Hexamethyl-p-terphenyl poly(benzimidazolium): a universal hydroxide-conducting polymer for energy conversion devices. *Energy & Environmental Science* **9**, 2130-2142 (2016).

19 Zhu, L. *et al.* Multication Side Chain Anion Exchange Membranes. *Macromolecules* **49**, 815-824 (2016).

20 Chen, N., Long, C., Li, Y., Lu, C. & Zhu, H. Ultrastable and High Ion-Conducting Polyelectrolyte Based on Six-Membered N-Spirocyclic Ammonium for Hydroxide Exchange Membrane Fuel Cell Applications. *ACS Appl Mater Interfaces* **10**, 15720-15732 (2018).
